# Supplementary material for: The final walk with preptin
Source: PLoS One. 2024 Sep 12;19(9):e0309726. doi: 10.1371/journal.pone.0309726 (PMC11392399; doi:10.1371/journal.pone.0309726)
Supplement: S1 Data — (DOCX) [file pone.0309726.s001.docx]

***THE FINAL WALK WITH PREPTIN?***

Lucie Mrázková^1,2^, Marta Lubos^1^, Jan Voldřich^1^, Erika Kužmová^1^, Denisa Zrubecká^1^, Petra Gwozdiaková^1^, Miloš Buděšínský^1^, Seiya Asai^1^, Aleš Marek^1^, Jan Pícha^1^, Michaela Tencerová^3^, Michaela Ferenčáková^3^, Glenda Alquicer Barrera^3^, Jakub Kaminský^1^, Jiří Jiráček^1^ and Lenka Žáková^1,^^[[1]](#footnote-1)^*

1. Institute of Organic Chemistry and Biochemistry of the Czech Academy of Sciences, Flemingovo nám. 2, 166 10 Prague, Czech Republic.
2. Department of Cell Biology, Faculty of Science, Charles University, Prague, Czech Republic
3. Institute of Physiology of the Czech Academy of Sciences, Vídeňská 1083, 142 00 Prague, Czech Republic.

**Content:**

# Pages 2-7 Synthesis of Amino acids

# Page 3 Scheme S1

# Page 5 Scheme S2

# Pages 7-8 HPLC traces of compounds IV-VI and X

# Page 7 Figures S1 and S2

# Page 8 Figures S3 and S4

# Pages 9-27 Analytical data (MS spectra and HPLC traces) for peptides 1-19

# Page 9 Peptide 1, Figures S5 and S6

# Page 10 Peptide 2, Figures S7 and S8

Page 11 Peptide **3**, **Figures S9** and **S10**

Page 12 Peptide **4**, **Figures S11** and **S12**

Page 13 Peptide **5**, **Figures S13** and **S14**

Page 14 Peptide **6**, **Figures S15** and **S16**

Page 15 Peptide **7**, **Figures S17** and **S18**

# Page 16 Peptide 8, Figures S19 and S20

# Page 17 Peptide 9, Figures S21 and S22

Page 18 Peptide **10**, **Figures S23** and **S24**

Page 19 Peptide **11**, **Figures S25** and **S26**

Page 20 Peptide **12**, **Figures S27** and **S28**

Page 21 Peptide **13**, **Figures S29** and **S30**

Page 22 Peptide **14, Figures S31** and **S32**

Page 23 Peptide **15**, **Figures S33** and **S34**

Page 24 Peptide **16**, **Figures S35** and **S36**

Page 25 Peptide **17**, **Figures S37** and **S38**

Page 26 Peptide **18**, **Figures S39** and **S40**

Page 27 Peptide **19, Figures S41 and S42**

Page 28 NMR configuration of stereochemistry of peptide **10**, **Table S1**

Page 29 NMR configuration of stereochemistry of peptide **11**, **Table S2**

Page 30 Details of the CD experiments with peptides **1-19**, **Table S3**

# Page 31 Experimental CD band wavelengths and intensities of 1-19, Table S4

Page 32 Secondary structure content for **1**-**19** according to the experimental CD, **Table S5**

# Page 33 Preparation of mouse [^125^I]-Preptin (peptide 14)

# Page 34 Typical saturation binding curves of [^125^I]-preptin 14 to different cells, Figure S43

Page 35 Stimulation of phosphorylation of Erk 1/2 by preptins **1**-**19, Figure S44**

Page 36 Stimulation of phosphorylation of PI3K p110α by preptins **1**-**19, Figure S45**

**Page 37** Representative western blot analysis of preptin derivatives **1-19, Figure S46**

# Page 37 References

# Synthesis of amino acids

Unless otherwise stated, the reagents and solvents used in this study were obtained from commercial suppliers (Penta chemicals, Lach-Ner, VWR Chemicals). TLC was performed on silica gel coated aluminium plates (Merck). The compounds were visualized by exposure to UV light at 254 nm, by ninhydrin spraying followed by heating (dark blue stain of BocHN or free NH_2_ species) or immersion of TLC plate to 1% aqueous solution of KMnO_4_ (yellow stain of oxidized thioether). Flash chromatography were carried out on silica gel (40-63 μm, VWR Chemicals). Analytical RP-HPLC was carried out using Method 1 [gradient: t = 0 min (20 % B), t = 30 min (100 % B), t = 31 min (20% B); solvent A was 0.1% TFA and solvent B 80 % AcCN, 0.1%TFA] at flow rate of 1 ml/min on column (EC 250/4.6 Nucleosil 100 – 5 C8, Macherey –Nagel). The eluted compounds were detected at 218 nm. Melting points were determined on a Boetius block and are uncorrected. ^1^H and ^13^C NMR spectra were acquired on Bruker AVANCE-600 spectrometer (^1^H at 600.13 MHz, ^13^C at 150.9 MHz) in CDCl_3_ or DMSO-d_6_ at 300 K. The 2D H H COSY, 2D H C HSQC and 2D H C HMBC spectra were recorded and used for the structural assignment proton and carbon signals. IR spectra were recorded on a Bruker IFS 55 Equinox apparatus. HRMS data were obtained on a FTMS mass spectrometer LTQ -orbitrap XL (Thermo Fisher, Bremen, Germany) in electrospray ionization mode. Optical power were measured in 2 ml cell on Autopol IV under Na lamp radiation.

Chemical purity of compounds **IV**, **V**, **VI** and **X** was confirmed by using RP-HPLC.

(*2R*) -2-{[(9H-fluoren-9-yl)methoxy]carbonyl}amino-3-mercaptopropanoic acid

Fmoc-L-Cys-OH **III**

(*2S*) -2-{[(9H-fluoren-9-yl)methoxy]carbonyl}amino-3-mercaptopropanoic acid

Fmoc-D-Cys-OH **IV**

Acids **III** and **IV** were prepared by Gongora-Benitez et al (1) reaction of (8.8 g ; 15 mmol) Fmoc-L-Cys(Trt)-OH **I** or (8.8 g ; 15 mmol) Fmoc-D-Cys(Trt)-OH **II** mediated by TFA, respectively (Scheme S1).

|  |
| --- |
| **Scheme S1.** Reagents, conditions, and yields: (a) TFA, TIPS, DCM, RT for 2 hours (87% for **III**, 92 % for **IV**); (b) allyl bromide, NaHCO_3_, TBAB, water and dioxane, rt for overnight (78% for **V**, 80% for **VI**). |

**III**. 4.5 g (87 %). Colourless solid. Spectra and physico-chemical characteristics of **III** were with good agreement of those described earlier^2^.

**IV**. 4.8 g (92 %). Colourless solid, m. p. 115 – 117 °C. R_f_ = 0.45 (ethyl acetate : MeOH : acetone : water = 6 : 1 : 1 : 0.5). $⍺_{20}^{D}$= +23.4 (c = 2 ; DMF). ^1^H NMR (600 MHz, DMSO; T = 25 °C): 2.50 bt (1H, *J* = 8.3 and 7.8 Hz, –SH), 2.75 dt (1H, *J* = 13.6 and 8.3 Hz) and 2.90 ddd (1H, *J* = 13.6, 7.8 and 4.3 Hz) (S–CH_2_–), 4.14 td (1H, *J* = 8.3 and 4.3 Hz, >CH–N), 4.24 bt (1H, *J* = 7.0 Hz, >CH–), 4.32 m (2H, O–CH_2_–), 7.33 m (2H, 2x Ar–H), 7.42 m (2H, 2x Ar–H), 7.695 d (1H, *J* = 8.3 Hz, >NH), 7.74 m (2H, 2x Ar–H), 7.89 m (2H, 2x Ar–H), 12.88 br (1H, COOH). ^13^C NMR (150.9 MHz, DMSO; T = 25 °C): 25.64 (S–CH_2_–), 46.83 (>CH–), 56.76 (>CH–N), 65.92 (O–CH_2_–), 120.31 (2x Ar =CH–), 125.47 (2x Ar =CH–), 127.28 (2x Ar =CH–), 127.84 (2x Ar =CH–), 140.92 (2x Ar =C<), 143.98 (Ar =C<), 143.99 (Ar =C<), 156.25 (O–CO–N), 172.06 (COOH). IR (KBr) υ_max_ (cm^-1^) 3314 m (NH); 1717 vs (C=O) carbamate, 1702 vs (C=O) acid; 1533 s (amide II); 2565 m (SH); 3065 m, 3039 m, 1611 w, 1579 vw, 1478 m, 1451 m, 1033 m, 758 s, 739 vs (fluorene). HRMS (ESI) calc for C_18_H_18_O_4_NS [M+H]^+^ 344.09511, found 344.09502.

(*2R*) -2-{[(9H-fluoren-9-yl)methoxy]carbonyl}amino-3-(allylsulfanyl) propanoic acid

Fmoc-L-Cys(allyl)-OH **V**

(*2S*) -2-{[(9H-fluoren-9-yl)methoxy]carbonyl}amino-3-(allylsulfanyl) propanoic acid

Fmoc-D-Cys(allyl)-OH **VI**

70 ml of aqueous solution of NaHCO_3_ (5 g ; 60 mmol) and TBAB (0.5 g ; 1.5 mmol) was added to **III** (5.2 g ; 15 mmol) and allyl bromide (1.6 ml ; 18 mmol) in 70 ml of ethyl acetate and biphasic system was vigorously stirred under argon atmosphere overnight. 10% citric acid was added until pH value of reaction mixture was about 3. Reaction mixture was transformed to a separatory funnel, organic phase was separated, and aqueous phase extracted with 2 x 100 ml of ethyl acetate. Combined organic layers were washed with 100 ml of water, 2 x 100 ml of brine and dried with Na_2_SO_4_. Evaporation of filtrate under reduced pressure gave pale brown oil, which was subjected flash chromatography on silica using a linear gradient of 1% AcOH/ ethyl acetate in toluene. Isolated light yellow oil was dissolved with heating in 20 ml of toluene and placed for 24 hours to -20 °C. 50 ml of hexane was added to resulting colourless gel and mixture was sonicated for 10 minutes in water bath. Flask with solid product was again placed at -20 °C for 24 hours. Precipitate was filtered off, washed with 50 ml of hexane and dried under deep vacuum.

**V**. Yield 4.5 g (78%). White solid, m. p. 64 - 67 °C. R_f_ = 0.65 (DCM : MeOH : conc. NH_4_OH = 75 : 22 : 3).$⍺_{20}^{D}$= -12.8 (c = 2 ; CH_2_Cl_2_). ^1^H NMR (600 MHz, DMSO; T = 25 °C): 2.70 dd (1H, *J* = 13.6 and 9.6 Hz) and 2.865 dd (1H, *J* = 13.6 and 4.6 Hz) (S–CH_2_–), 3.17 bd (2H, *J* = 7.1, 1.5 and 1.5 Hz, S–CH_2_–), 4.13 ddd (1H, *J* = 8.2, 9.6 and 4.6 Hz, >CH–N), 4.235 bt (1H, *J* = 7.0 Hz, >CH–), 4.30 m (2H, O–CH_2_–), 5.08 dq (1H, *J* = 10.0 and 1.5 Hz) and 5.125 dq, *J* = 17.0 and 1.5 Hz) (=CH_2_), 5.75 ddt (1H, *J* = 17.0, 10.0 and 7.1 Hz, =CH–), 7.33 m (2H, 2x Ar–H), 7.42 m (2H, 2x Ar–H), 7.74 m (2H, 2x Ar–H), 7.755 d (1H, *J* = 8.2 Hz, >NH), 7.89 m (2H, 2x Ar–H), 12.84 br (1H, COOH). ^13^C NMR (150.9 MHz, DMSO; T = 25 °C): 31.71 (S–CH_2_–), 34.18 (S–CH_2_–), 46.82 (>CH–), 53.92 (>CH–N), 65.94 (O–CH_2_–), 117.55 (=CH_2_), 120.30 (2x Ar =CH–), 125.48 (Ar =CH–), 125.49 (Ar =CH–), 127.27 (2x Ar =CH–), 127.84 (2x Ar =CH–), 134.48 (=CH–), 140.91 (Ar =C<), 140.92 (Ar =C<), 143.98 (Ar =C<), 144.00 (Ar =C<), 156.21 (O–CO–N), 172.52 (COOH). IR (KBr) υ_max_ (cm^-1^) 3324 m (NH); 1711 vs (C=O) acid, 1694 vs (C=O) carbamate; 1534 s (amide II); 1635 m (C=C); 3066 m, 3041 m, 1610 w, 1579 w, 1478 m, 1451 s, 758 s, 739 vs (fluorene). HRMS (ESI) calc for C_21_H_20_O_4_NS [M–H]^–^ 382.11185, found 382.11169.

Compound **VI** was prepared reaction of (5 g ; 14.6 mmol) **IV**, (1.5 ml ; 17.7 mmol) allyl bromide, (4.9 g ; 58.2 mmol) NaHCO_3_ and (0.47 g ; 1.46 mmol) TBAB using the procedure used for **V**.

**VI**. Yield 4.5 g (80%). White solid, m. p. 63 - 65 °C. R_f_ = 0.65 (DCM : MeOH : conc. NH_4_OH = 75 : 22 : 3).$⍺_{20}^{D}$= +12.4 (c = 2 ; CH_2_Cl_2_). ^1^H NMR (600 MHz, DMSO; T = 25 °C): 2.695 dd (1H, *J* = 13.7 and 9.6 Hz) and 2.85 dd (1H, *J* = 13.7 and 4.7 Hz) (S–CH_2_–), 3.17 bd (2H, *J* = 7.1, 1.5 and 1.5 Hz, S–CH_2_–), 4.13 ddd (1H, *J* = 8.2, 9.6 and 4.7 Hz, >CH–N), 4.235 bt (1H, *J* = 7.0 Hz, >CH–), 4.30 m (2H, O–CH_2_–), 5.08 ddt (1H, *J* = 10.0, 1.8 and 1.5 Hz) and 5.125 ddt, *J* = 17.0, 1.8 and 1.5 Hz) (=CH_2_), 5.75 ddt (1H, *J* = 17.0, 10.0 and 7.1 Hz, =CH–), 7.33 m (2H, 2x Ar–H), 7.42 m (2H, 2x Ar–H), 7.74 m (2H, 2x Ar–H), 7.75 d (1H, *J* = 8.2 Hz, >NH), 7.89 m (2H, 2x Ar–H), 12.84 br (1H, COOH). ^13^C NMR (150.9 MHz, DMSO; T = 25 °C): 31.70 (S–CH_2_–), 34.17 (S–CH_2_–), 46.81 (>CH–), 53.91 (>CH–N), 65.92 (O–CH_2_–), 117.54 (=CH_2_), 120.29 (2x Ar =CH–), 125.47 (Ar=CH–), 125.48 (Ar =CH–), 127.26 (2x Ar =CH–), 127.83 (2x Ar =CH–), 134.48 (=CH–), 140.90 (Ar =C<), 140.91 (Ar =C<), 143.97 (Ar =C<), 143.99 (Ar =C<), 156.19 (O–CO–N), 172.51 (COOH). IR (KBr) υ_max_ (cm^-1^) 3325 s (NH); 1709 vs (C=O) acid, 1692 vs (C=O) carbamate; 1534 s (amide II); 1635 m (C=C); 3066 m, 3041 m, 1611 w, 1579 w, 1478 m, 1451 s, 758 s, 740 vs (fluorene). HRMS (ESI) calc for C_21_H_20_O_4_NS [M–H]^–^ 382.11185, found 382.11201.

Protected asparagine **X** was synthesized as shown in Scheme S2. Unlike in our protocol described earlier (2), intermediate **IX** was isolated and fully characterized. Another change was a finding, that product **X** has a high purity (RP-HPLC 95% rel.) after a classical work-up of the reaction mixture and therefore flash chromatography isn´t necessary. The material prepared in this way enables SPSS without any restrictions (3).

|  |
| --- |
| **Scheme S2.** Reagents, conditions and yields: (a) DIC, DMAP, triethylcarbinol, DCM 0 °C 1 h, then rt overnight (79%); (b) 10% Pd/C, H_2_, 15 psi, methanol 24 h (88%); (c) Fmoc-Osu, NaHCO_3_, water and dioxane, 0 °C 1 h, then rt overnight (90%). |

*N*-benzyloxycarbonylaspartic acid 1-benzyl-4-((3-ethyl)pent-3-yl) ester

(Z-Asp(OEpe)-OBzl) **VIII**

Ester was prepared reaction Z-Asp-OH (10 g ; 28 mmol) **VII** , triethylcarbinol (11.8 ml ; 84 mmol), DIC (5.2 ml ; 33.6 mmol) and DMAP (0.68 g ; 5 mmol) using protocol published earlier. Yield 10 g (79%). Spectra were identical to those already depicted in our previous publication (2).

Aspartic acid 4-((3-ethyl)pent-3-yl) ester

(Asp(OEpe)-OH) **IX**

Intermediate (8.4 g ; 18,4 mmol) **VIII** was dissolved in 200 ml of methanol and then 500 mg 10% Pd/C was added. The mixture was vigorously stirred and allowed to react under atmosphere of hydrogen (15 psi) at rt for 24 hours. TLC analysis (toluen : ethyl acetate 80 : 20) revealed complete disappearance of the starting compound. The catalyst was filtered off through Celite and filter pad was washed with 250 ml of methanol. Filtrate was concentrated under reduced pressure on rotary evaporator. The resulting light brown oil was dissolved with heating (60 °C) in minimal volume of methanol and after cooling, ether was dropwised added until solid material precipitated. Flask was placed to -20 °C overnight, crystals were filtered with using of Büchner funnel, washed with 50 ml of chilled ether and dried under deep vacuum. Yield 3.7 g (88%). White solid, m. p. 150 °C softening, 195 °C decay. R_f_ = 0.69 (IPA : water : conc. NH_4_OH = 7 : 4 : 1)$. ⍺_{20}^{D}$= +44.8 ( c = 2 ; glacial acetic acid). ^1^H NMR (600 MHz, DMSO; T = 25 °C): 0.78 t (9H, *J* = 7.4 Hz, 3x -CH_3_), 1.76 q (6H, *J* = 7.4 Hz, 3x -CH_2_-), 2.52 dd (1H, *J* = 17.2 and 7.4 Hz, and 2.79 dd (1H, *J* = 17.2 and 4.8 Hz, CO-CH_2_-), 3.39 dd (1H, *J* = 7.4 and 4.8 Hz, >CH-N). ^13^C NMR (150.9 MHz, DMSO; T = 25 °C): 7.65 (3x -CH_3_), 26.65 (3x -CH_2_-), 36.61 (CO-CH_2_-), 50.46 (>CH-N), 88.18 (>C<), 168.88 (O-CO-), 170.16 (COOH). IR (KBr) υ_max_ (cm^-1^) 1726 vs (C=O, ester); 1706 vs (C=O, acid); 1595 m, 1397 m (COO^-^); 1238 s (C-O-C); 1641 vs (NH_2_); 1526 s ($\mathrm{NH}_{3}^{+}$); 2974 vs, 2884 s, 1380 s (CH_3_); 2945 s (CH_2_); 1167 m (CEt_3_). HRMS (ESI) calc for C_11_H_20_O_4_N [M-H]^-^ 230.13978, found 230.13963.

*N*-((9*H*-fluoren-9-ylmethyloxy)carbonyl)aspartic acid-4-((3-ethyl)pent-3-yl) ester

(Fmoc-Asp(OEpe)-OBzl) **X**

Acid **IX** was suspended in 50 ml solution of NaHCO_3_ (3.4 g ; 14.7 mmol) and under ice-cooling Fmoc-Osu (5 g ; 14.7 mmol) was added dropwise in 50 ml of dioxane. Stirring continued 1 hour at 0 °C and then at rt overnight. Flask was again immersed to ice-cooling bath and 0.1 M HCl was added until pH ~ 1 - 2 was reached. Reaction mixture was extracted with 4 x 100 ml of ethyl acetate, combined organic phases were washed with 1 x 100 ml of water, 2 x 100 ml of brine, and dried with Na_2_SO_4_. The filtrate was evaporated under reduced pressure, light yellow oil was dissolved in mixture water – AcCN (1 : 1), resulting solution was first frozen at -80 °C and then lyophilized. Process of dissolution and lyophilization was repeated twice more. Yield 6 g (90%). Semisolid. Spectra were identical to those already depicted in our paper(2).

| 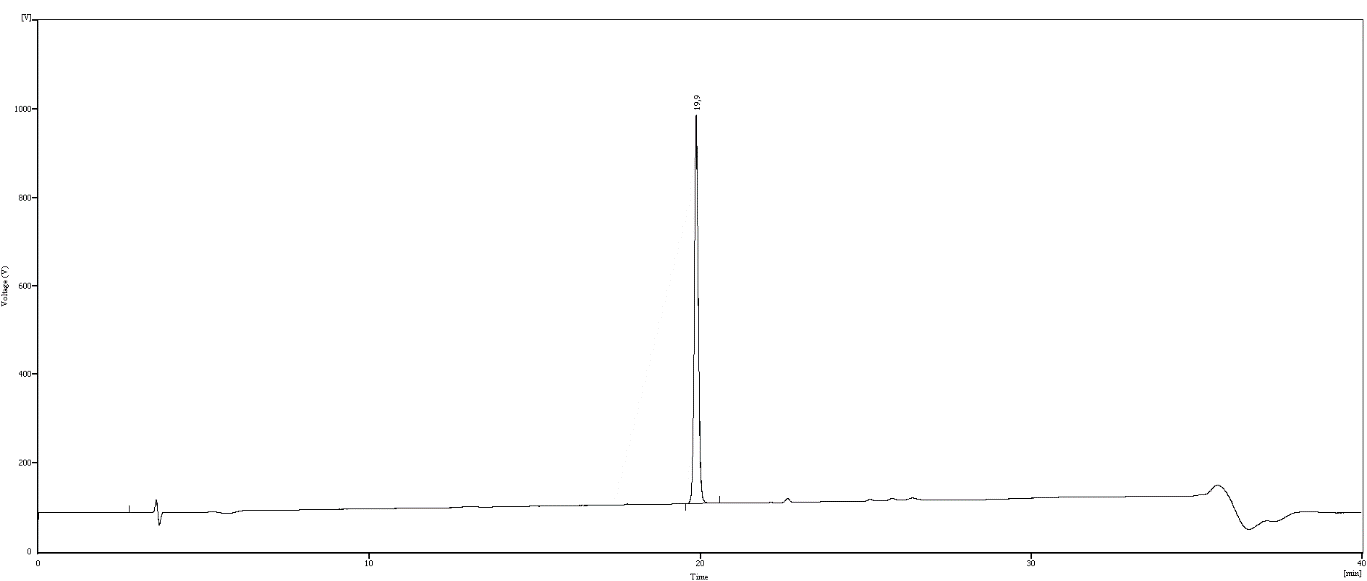 |
| --- |
| **Figure S1.** RP-HPLC profile of **IV** using a gradient from Method 1. |

| 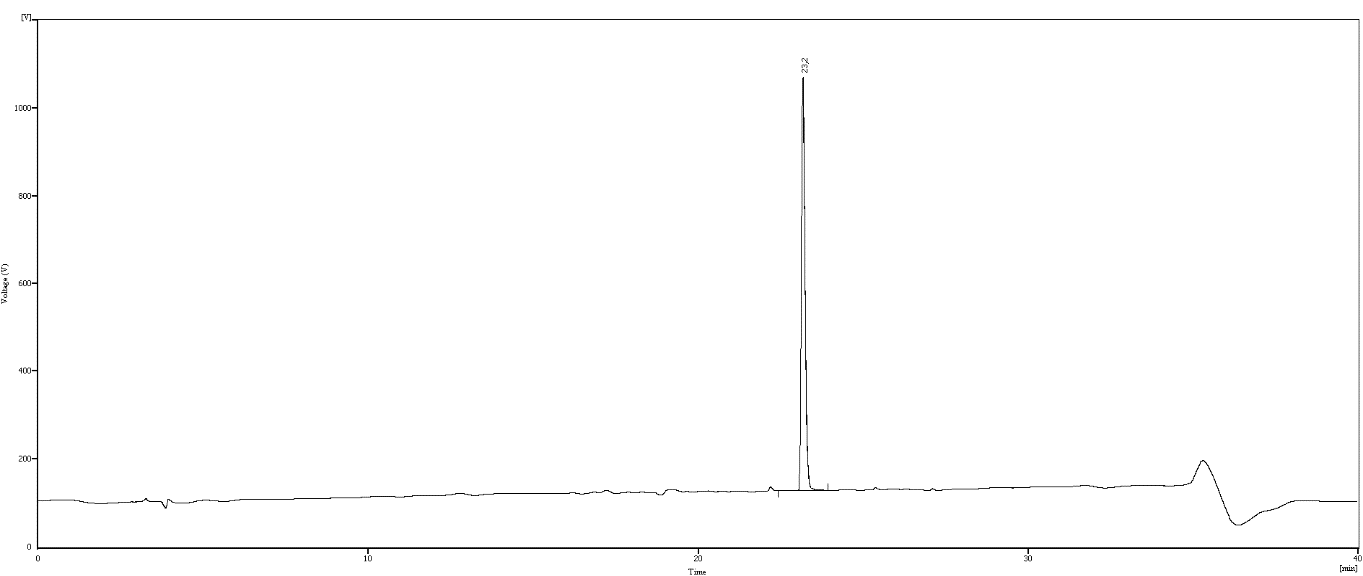 |
| --- |
| **Figure S2.** RP-HPLC profile of **V** using a gradient from Method 1. |

| 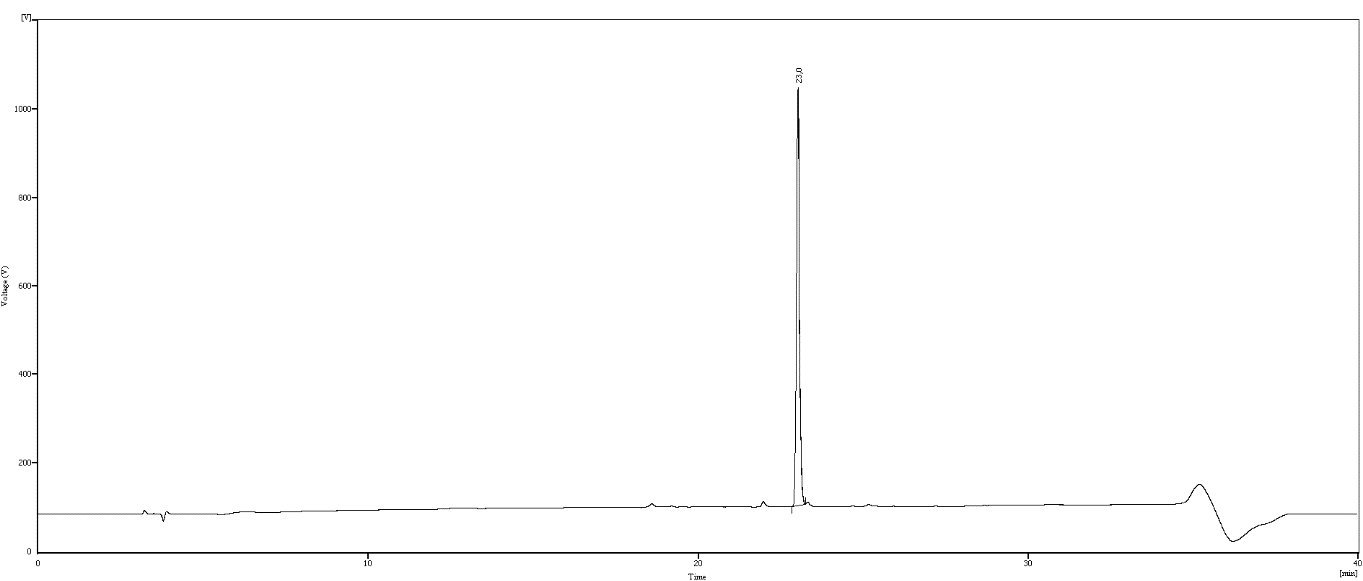 |
| --- |
| **Figure S3.** RP-HPLC profile of **VI** using a gradient from Method 1.   \| 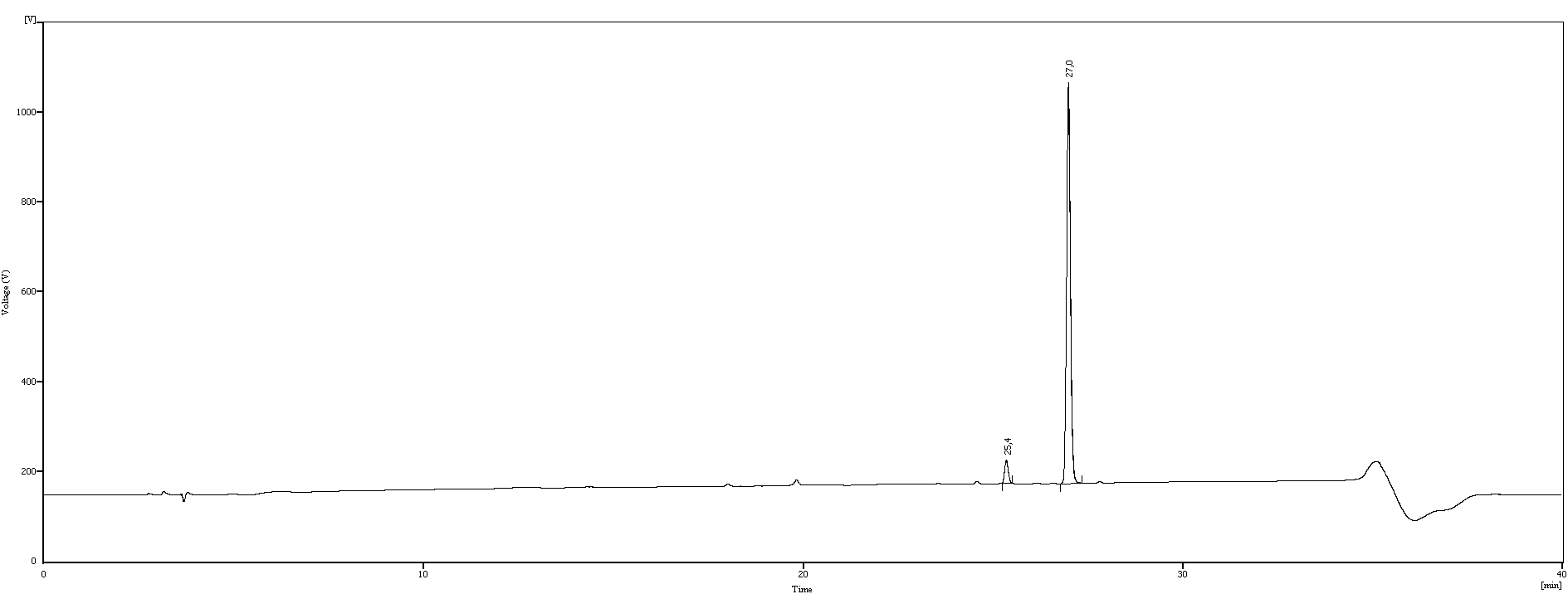 \| \| --- \| \| **Figure S4.** RP-HPLC profile of **X** using a gradient from Method 1. \| |

**Peptide 1** – Human preptin

DVSTPPTVLPDNFPRYPVGKFFQYDTWKQSTQRL


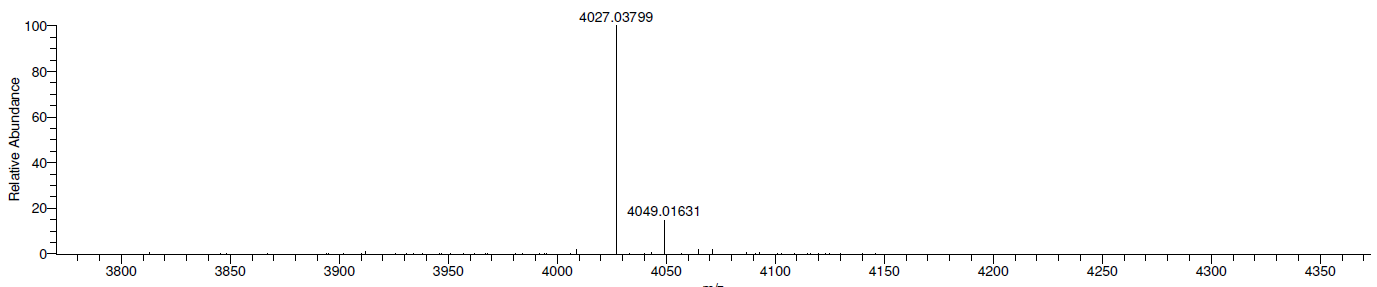


**Figure S5.** Deconvoluted mass spectrum of peptide **1**. Measured in positive ESI mode. The detected molecular weight was 4027.03799 (+2H^+^ + 2Na^+^). Chemical Formula: C183H270N50O51, Exact Mass: 3984.0071, Molecular Weight: 3986.4720.


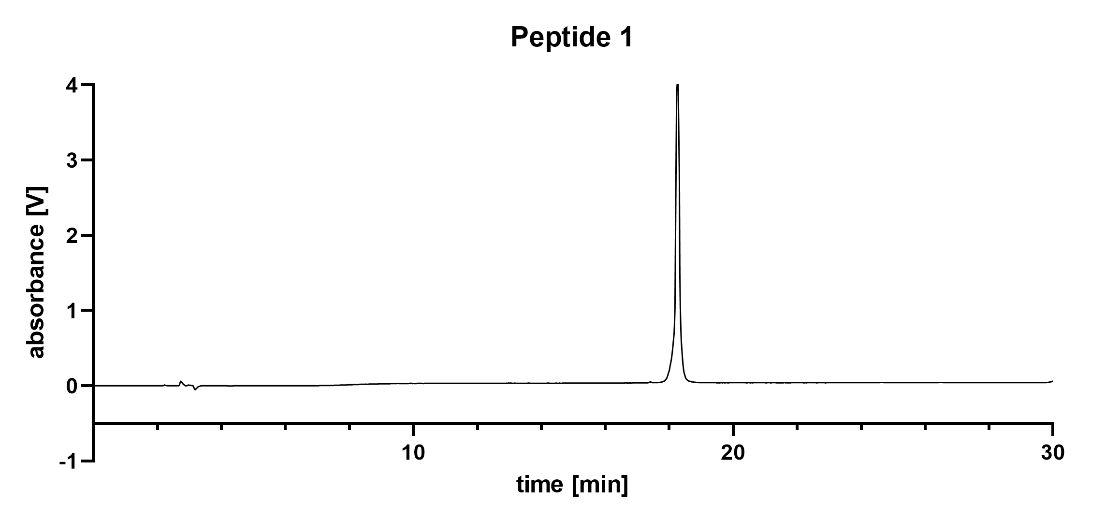


**Figure S6.** Analytical chromatogram of peptide **1**.

**Peptide 2** – Human preptin fragment

DVSTPPTVLPDNFPRY


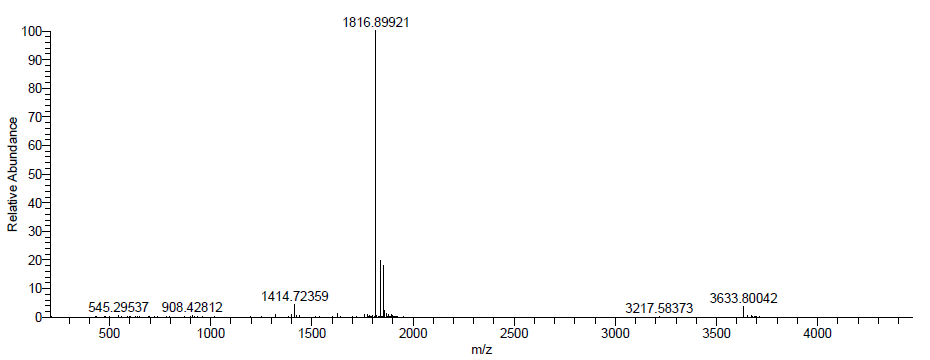


**Figure S7.** Deconvoluted mass spectrum of peptide **2**. Measured in positive ESI mode. The detected molecular weight was 1816.89921. Chemical Formula: C183H124N20O26, Exact Mass: 1816.8996, Molecular Weight: 1818.0190.


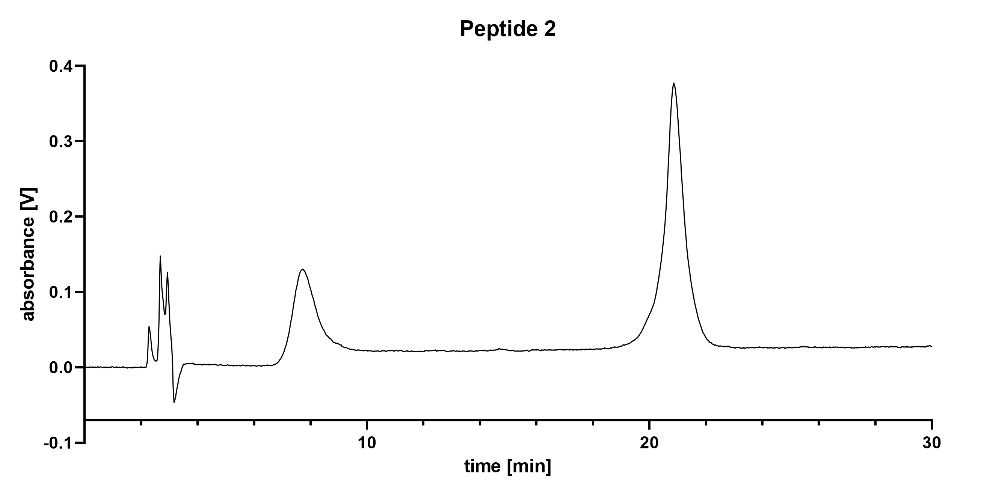


**Figure S8.** Analytical chromatogram of peptide **2**. The compound is eluted at 20.5 min. The apparent peak at 9.5 min is due to a rapid change in the acetonitrile content of the eluent from 10 to 35%.

**Peptide 3** – Human preptin fragment

PVGKFFQYDTWKQSTQRL


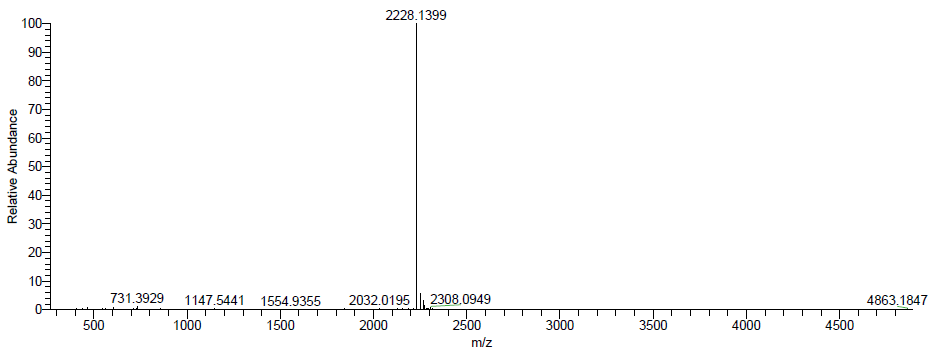


**Figure S9.** Deconvoluted mass spectrum of peptide **3**. Measured in positive ESI mode. The detected molecular weight was 2228.1399. Chemical Formula: C104H153N27O28, Exact Mass: 2228.1378, Molecular Weight: 2229.5290.


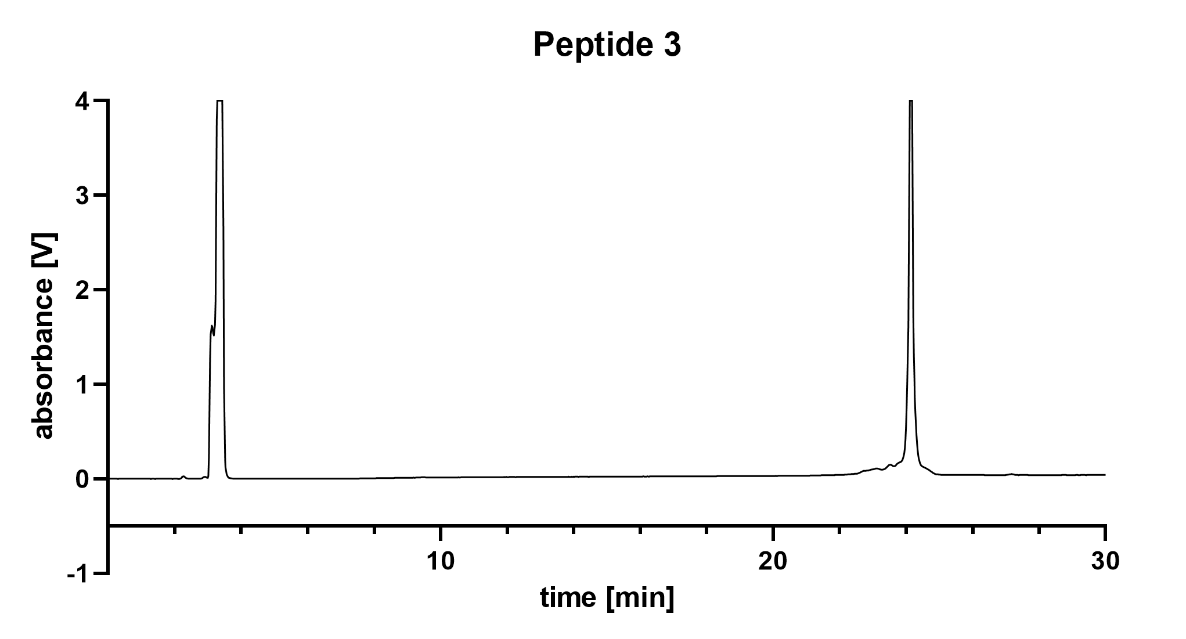


**Figure S10.** Analytical chromatogram of peptide **3**.

**Peptide 4** – Human preptin fragment

DVSTPPTVLPDNFPRY-amide


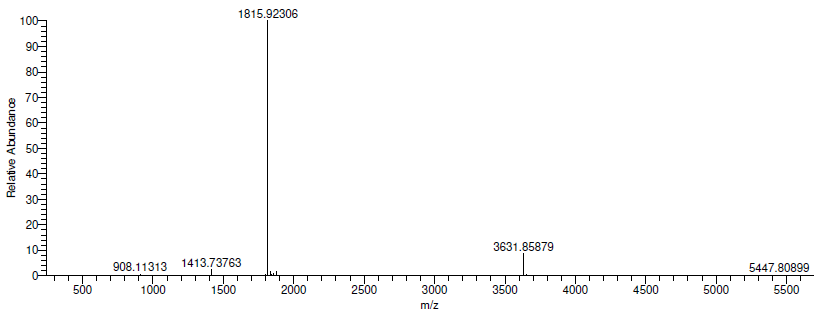


**Figure S11.** Deconvoluted mass spectrum of peptide **4**. Measured by in ESI mode. The detected molecular weight was 1815.9231. Chemical Formula: C83H125N21O25, Exact Mass: 1815.9155, Molecular Weight: 1817.0350.


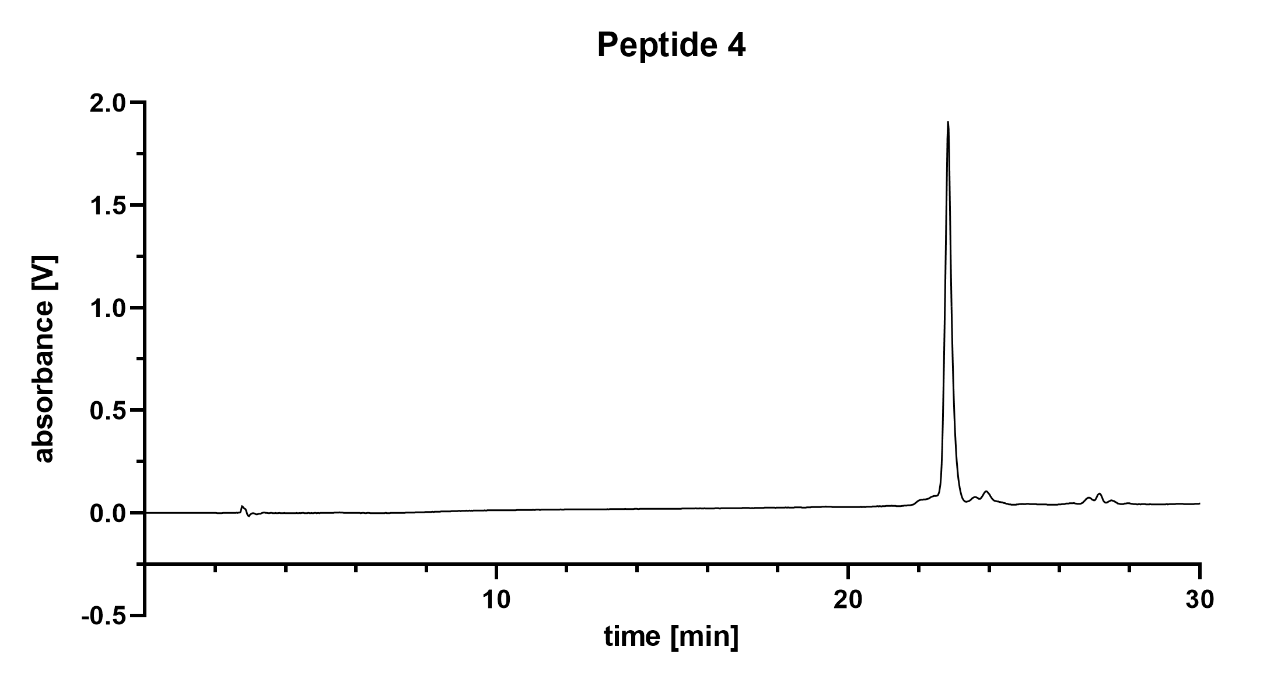


**Figure S12.** Analytical chromatogram of peptide **4**.

**Peptide 5** – Human preptin with D-Phe21

DVSTPPTVLPDNFPRYPVGK*F*FQYDTWKQSTQRL

**Figure S13.** Deconvoluted mass spectrum of peptide **5**. Measured in positive ESI mode. The detected molecular weight was 4027.03 (+2H^+^ + 2Na^+^). Chemical Formula: C183H270N50O51, Exact Mass: 3984.0071, Molecular Weight: 3986.4720.

**Figure S14.** Analytical chromatogram of peptide **5**.

**Peptide 6 –** Positions 16 and 20 both have ***S*** configuration.

DVSTPPTVLPDNFPRX^(16)^PVGX^(20)^FFQYDTWKQSTQRL-amide

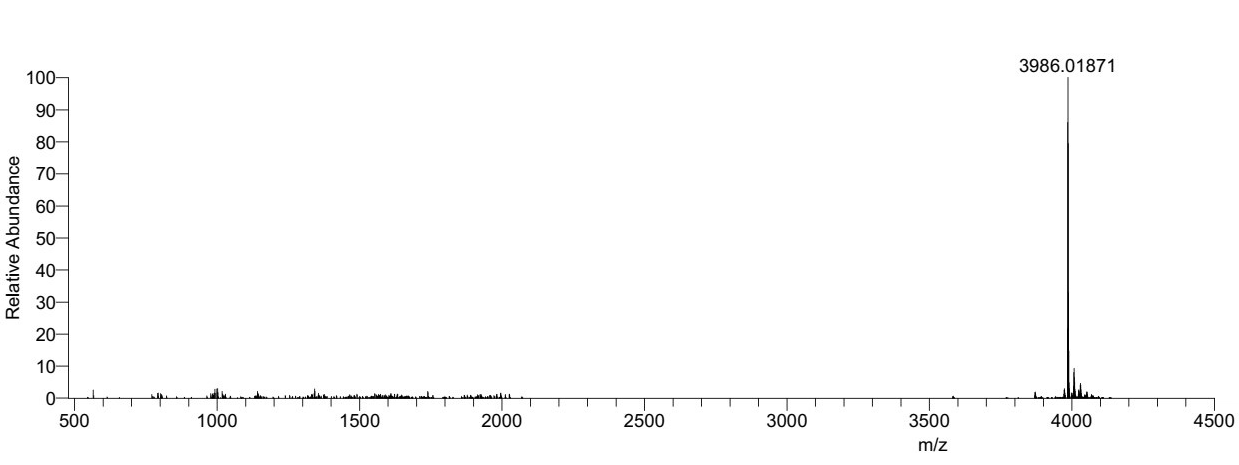


**Figure S15.** Mass spectrum of peptide **6**. Measured in ESI mode. The detected molecular weight was 3986.0187. Chemical Formula: C183H270N50O51, Exact Mass: 3984.0071, Molecular Weight: 3986.4720.

**Figure S16.** Analytical chromatogram of peptide **6**.

**Peptide 7 –** Position 16 has ***R*** and position 20 has ***S*** configuration.

DVSTPPTVLPDNFPRX^(16)^PVGX^(20)^FFQYDTWKQSTQRL-NH_2_

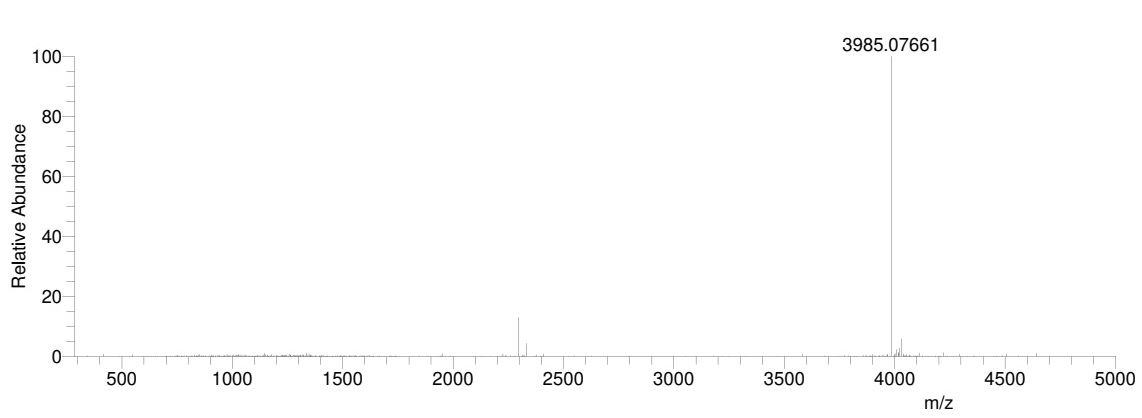


**Figure S17.** Deconvoluted mass spectrum of peptide **7**. Measured in ESI mode. Detected molecular weight was 3985.0766. Chemical Formula: C186H277N47O51, Exact Mass: 3985.0527, Molecular Weight: 3987.5400.

**Figure S18.** Analytical chromatogram of peptide **7**.

**Peptide 8 –** Position 13 has ***R*** and position 20 has ***S*** configuration.

DVSTPPTVLPDNX^(13)^PRYPVGX^(20)^FFQYDTWKQSTQRL-NH_2_

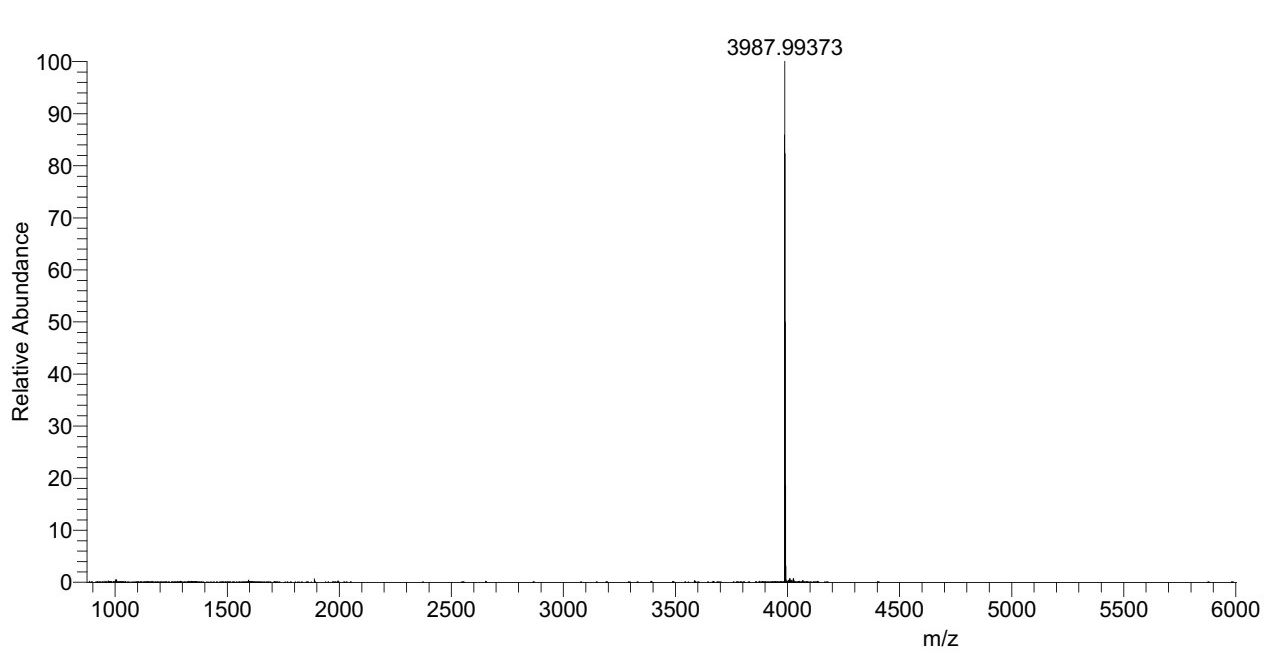


**Figure S19.** Mass spectrum of peptide **8**. Measured in ESI mode. Detected molecular weight was 3987.9937. Chemical Formula: C182H268N50O52, Exact Mass: 3985.9864, Molecular Weight: 3988.4440.

**Figure S20.** Purification chromatogram of peptide **8**.

**Peptide 9 –** Human preptin fragment, precursor for metathesis. Position 3 has ***R*** and position 6 has ***R*** configuration.

TW*X*^(3)^QST*X*^(6)^RL

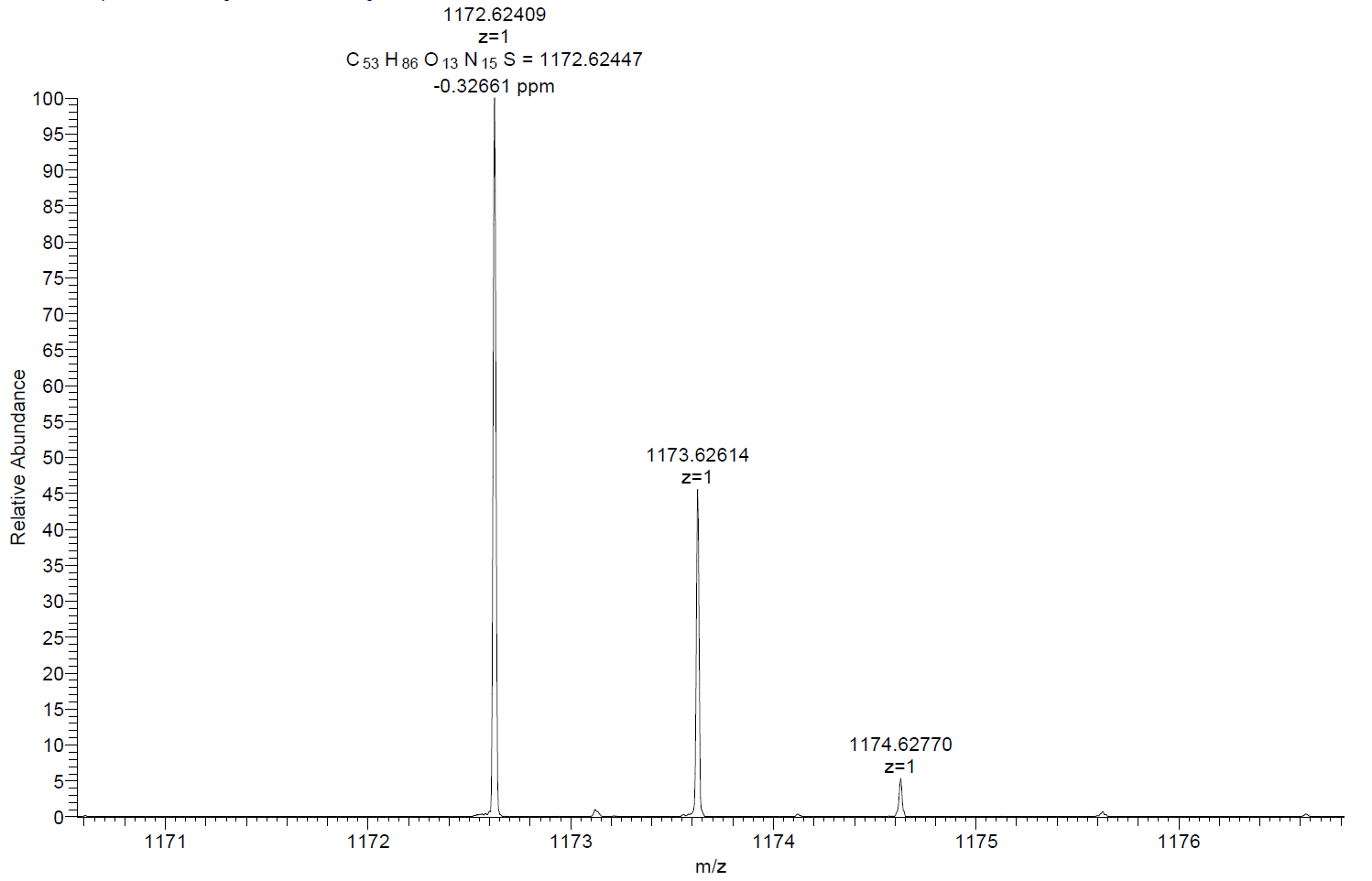


**Figure S21.** Deconvoluted mass spectrum of peptide **9**. Measured by in ESI mode. The detected molecular weight was 1172.6241. Chemical Formula: C53H84N14O14S, Exact Mass: 1172.6012, Molecular Weight: 1173.3990.


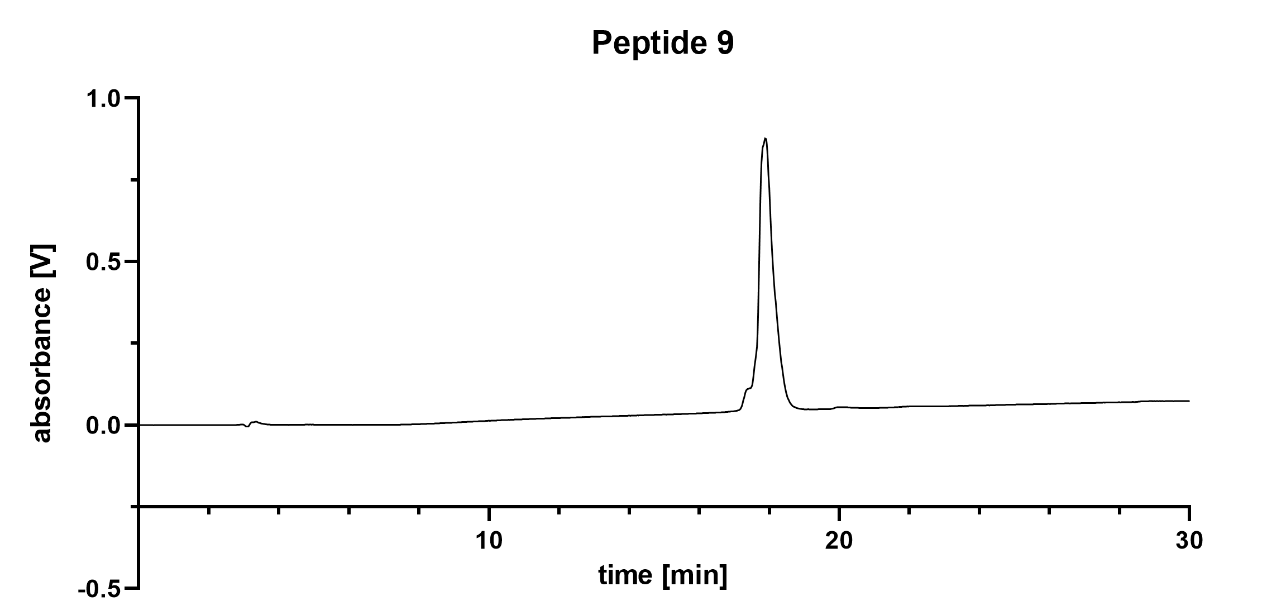


**Figure S22.** Analytical chromatogram of peptide **9**.

**Peptide 10 –** Human preptin fragment with a dicarba bridge. Position 3 has ***R*** and 6 have **R** configuration.

TW*X*^(3)^QST*X*^(6)^RL

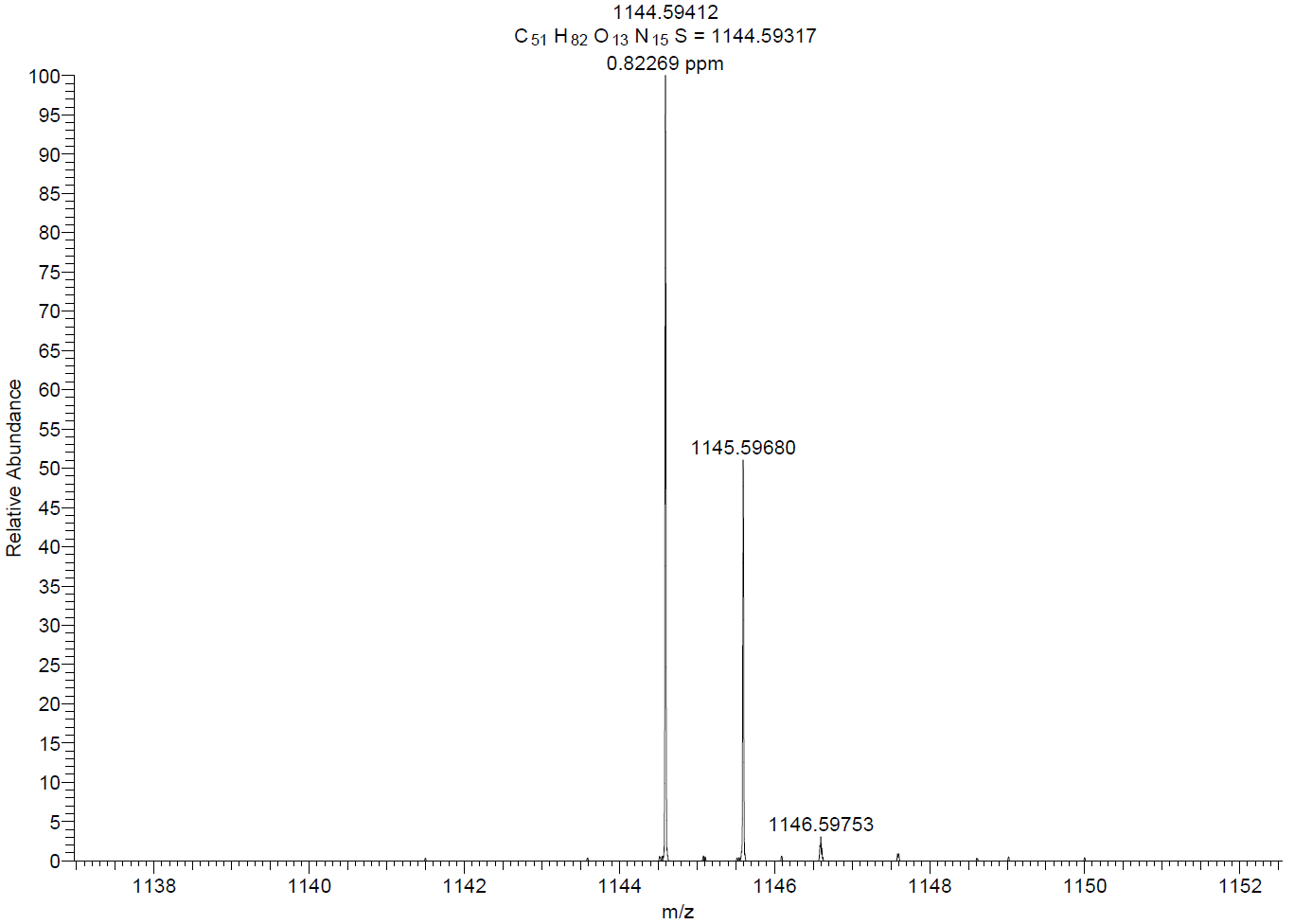


**Figure S23.** Deconvoluted mass spectrum of peptide **10**. Measured in positive ESI mode. The detected molecular weight was 1144.5941. Chemical Formula: C51H80N14O14S, Exact Mass: 1144.5699, Molecular Weight: 1145.3450.


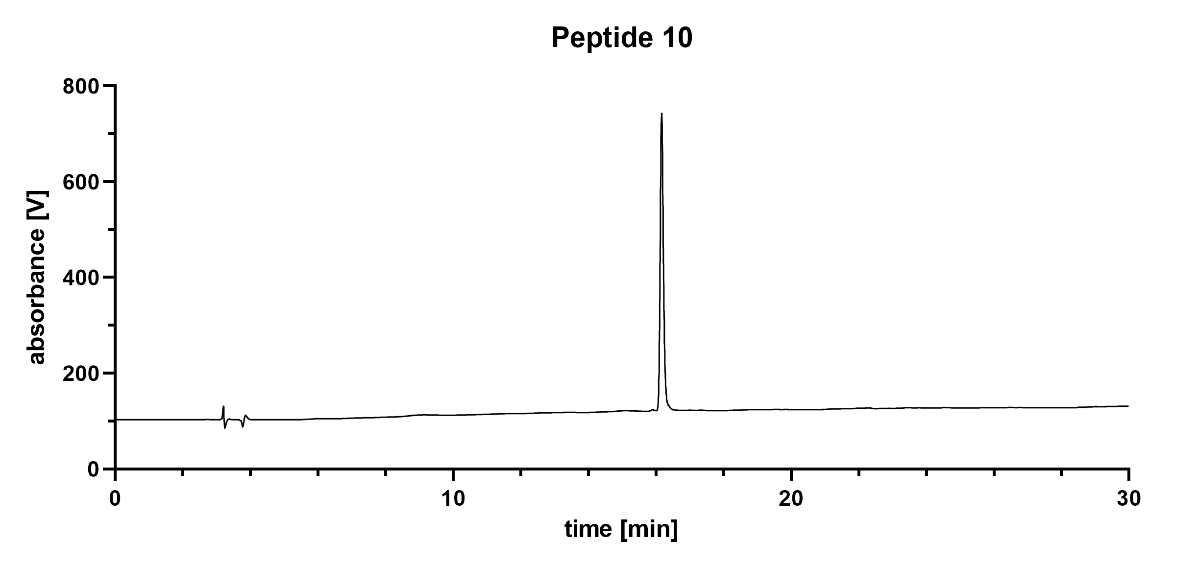


**Figure S24.** Analytical chromatogram of peptide **10**.

**Peptide 11 –** Human preptin fragment with a dicarba bridge. Position 3 has ***R*** and position 6 has ***S*** configuration.

TW*X*^(3)^QSTX^(6)^RL

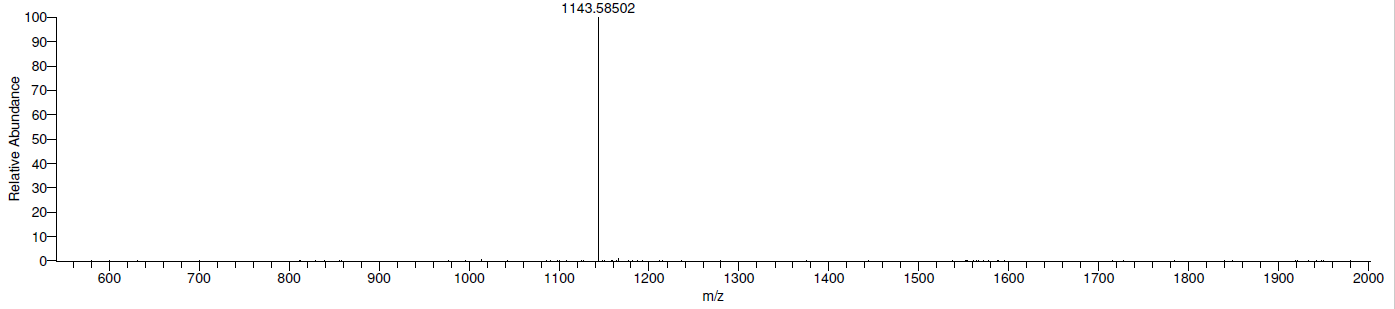


**Figure S25.** Deconvoluted mass spectrum of peptide **11**. Measured in positive ESI mode. The detected molecular weight was 1143.58502. Chemical Formula: C51H80N14O14S, Exact Mass: 1144.5699, Molecular Weight: 1145.3450.


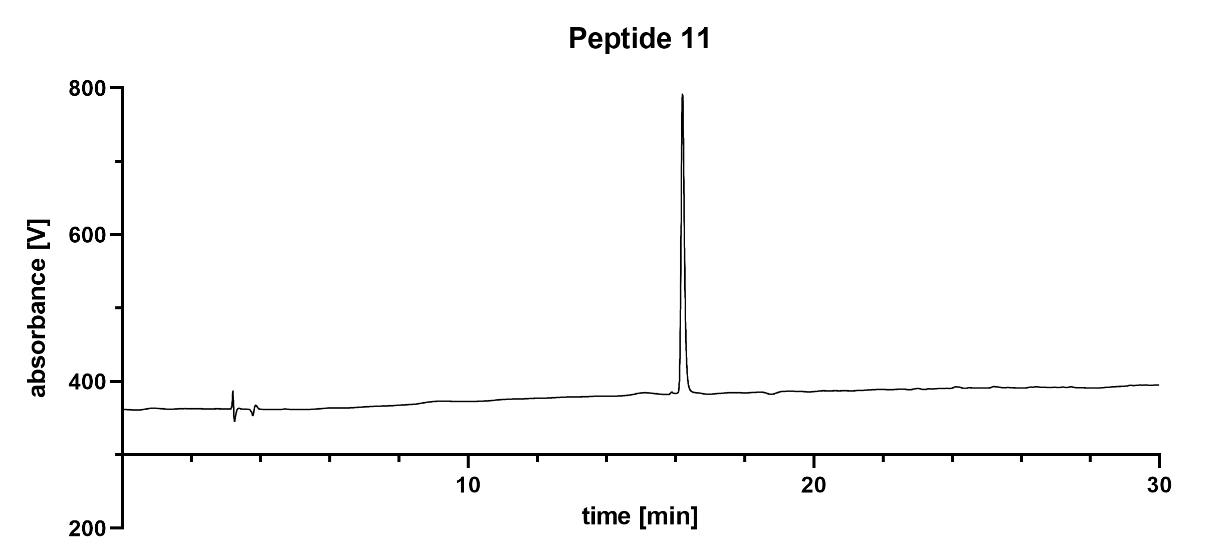


**Figure S26.** Analytical chromatogram of peptide **11**.

**Peptide 12 –** Human preptin fragment.

WKQSTQRL-amide


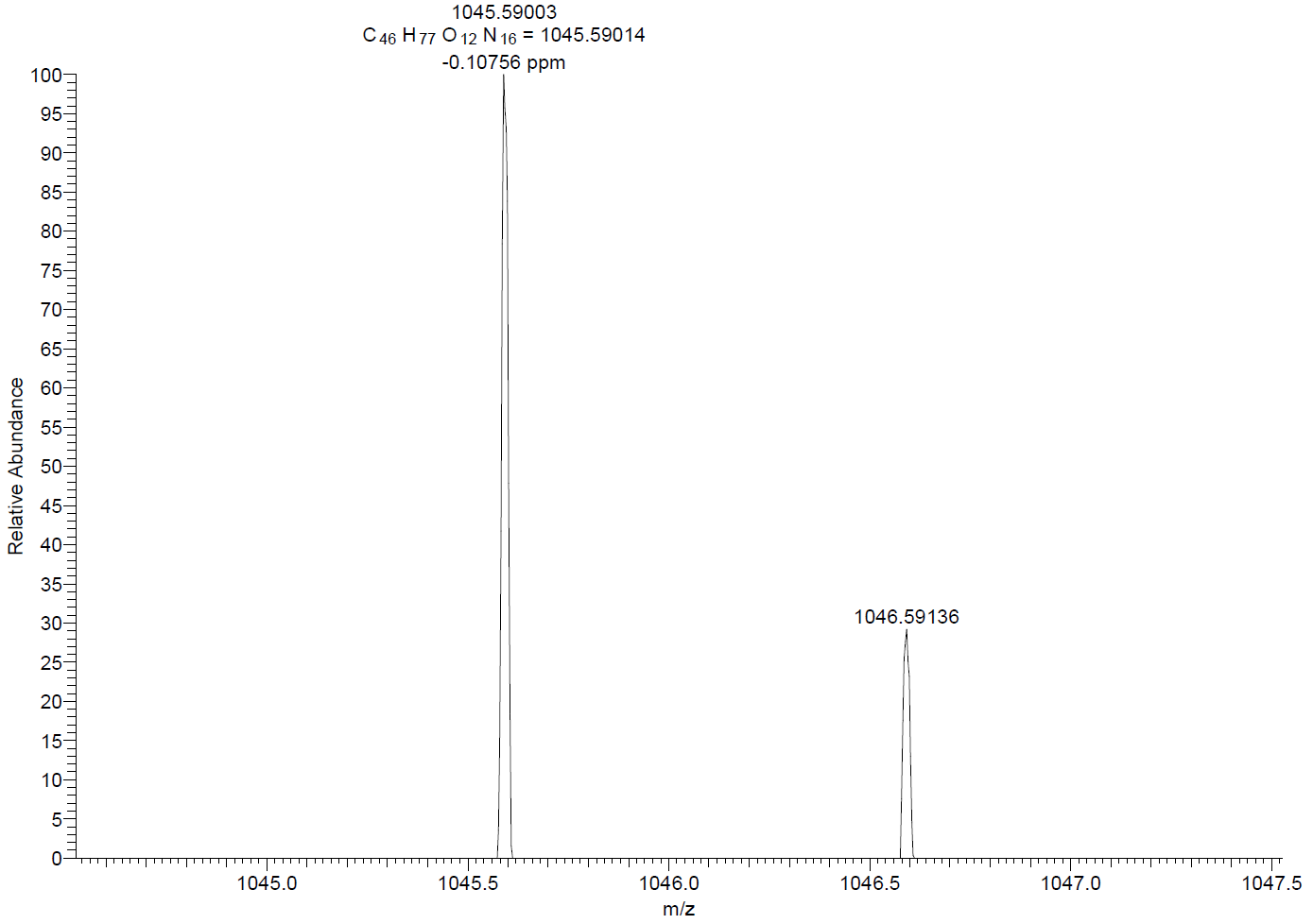


**Figure S27.** Mass spectrum of peptide **12**. Measured in positive ESI mode. The detected molecular weight was 1045.5900 (MH+). Chemical Formula: C46H760N16O12, Exact Mass: 1044.5829, Molecular Weight: 1045,2140.


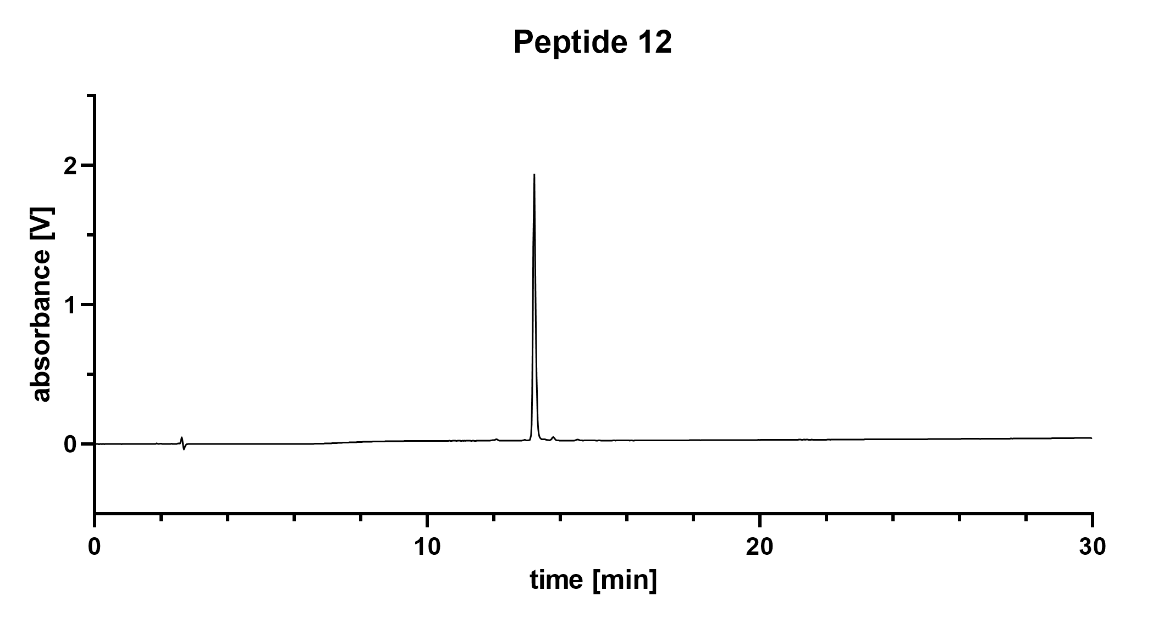


**Figure S28.** Analytical chromatogram of peptide **12**.

**Peptide 13 –** Human preptin fragment.

TWKQSTQRL-amide


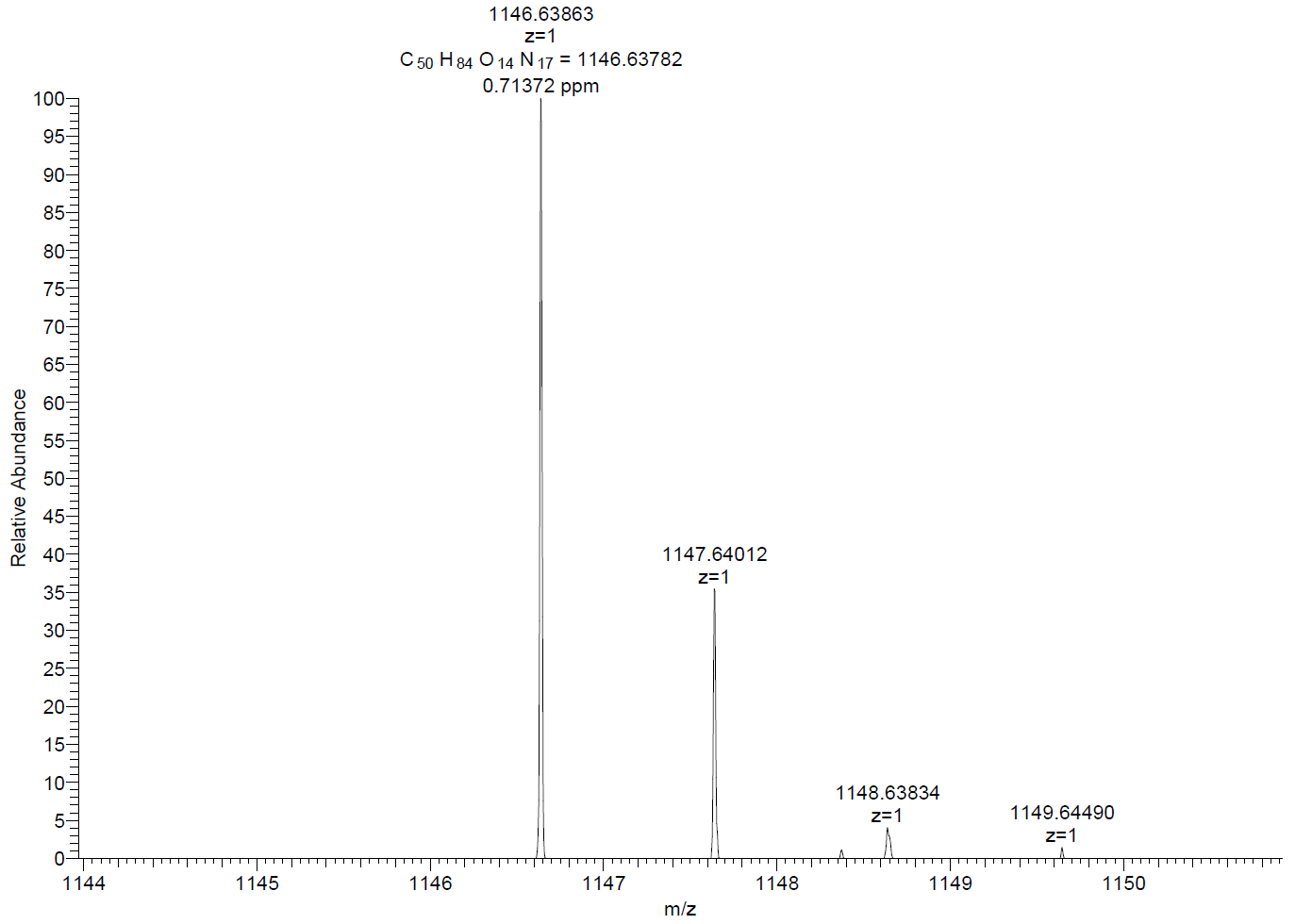


**Figure S29.** Deconvoluted mass spectrum of peptide **13**. Measured in positive ESI mode. The detected molecular weight was 1146.6386. Chemical Formula: C50H83N17O14, Exact Mass: 1145.6305, Molecular Weight: 1146.3090.


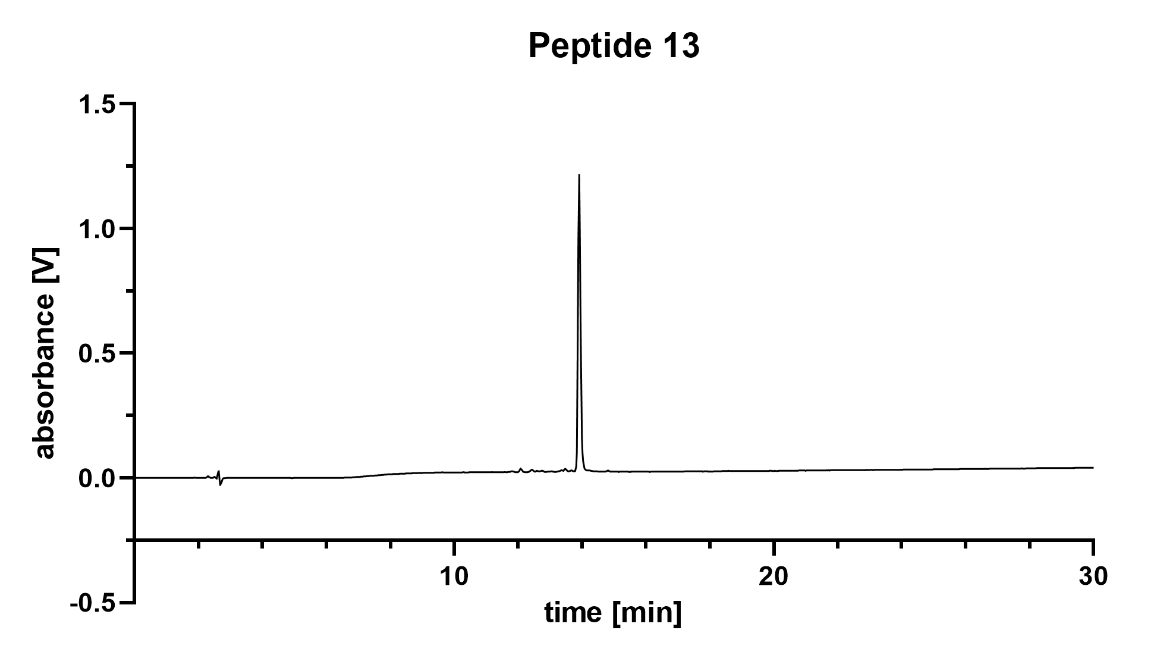


**Figure S30.** Analytical chromatogram of peptide **13**.

**Peptide 14 –** Mouse preptin

DVSTSQAVLPDDFPRYPVGKFFQYDTWRQSAGRL


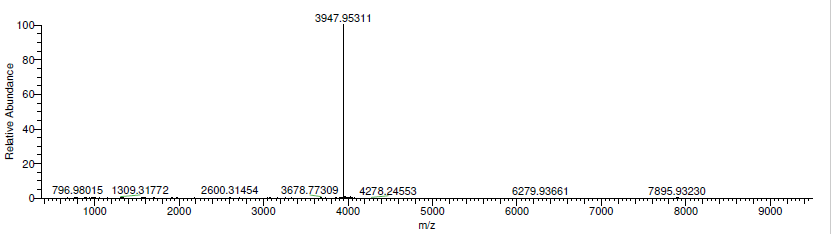


**Figure S31.** Mass spectrum of peptide **14**. Measured in positive ESI mode. The detected molecular weight was 3947.9531 (MH+). Chemical Formula: C180H264N48O53, Exact Mass: 3945.9438, Molecular Weight: 3948.3750.


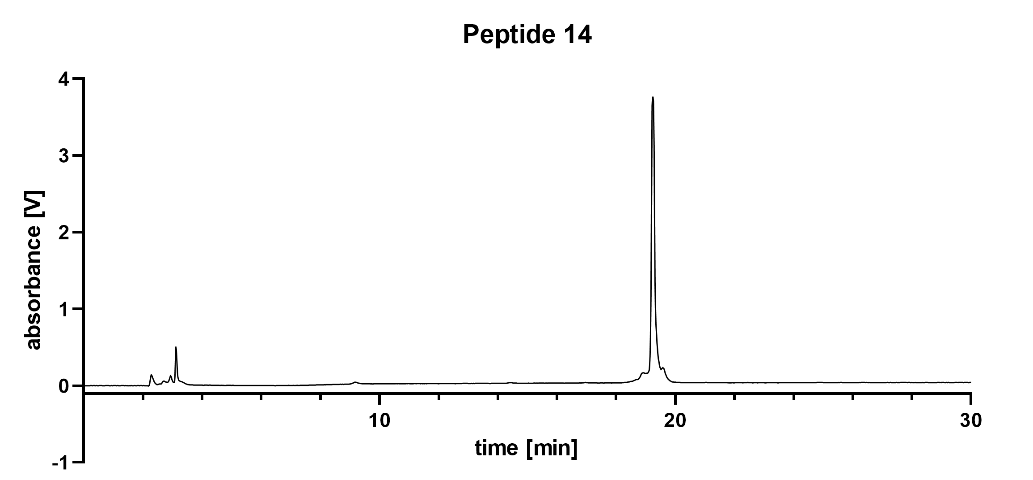


**Figure S32.** Analytical chromatogram of peptide **14**.

**Peptide 15 –** Mouse preptin with Aib (Z) at position 20.

DVSTSQAVLPDDFPRYPVGZFFQYDTWRQSAGRL

**Figure S33.** Deconvoluted mass spectrum of peptide **15**. Measured in positive ESI mode. The detected molecular weight was 3902.9176. Chemical Formula: C178H259N47O53, Exact Mass: 3902.9016, Molecular Weight: 3905.3060.

**Figure S34.** Analytical chromatogram of peptide **15**.

**Peptide 16 –** Mouse preptin fragment.

DVSTSQAVLPDDFPRY

**Figure S35.** Deconvoluted mass spectrum of peptide **16**. Measured in positive ESI mode. The detected molecular weight was 1808.8590. Chemical Formula: C80H120N20O28, Exact Mass: 1808.8581, Molecular Weight: 1809.9520.

Figure **S36**. Analytical chromatogram of peptide **16**.

**Peptide 17 –** Mouse preptin fragment.

DVTTSQAVLPDDFPRY

**Figure S37.** Deconvoluted mass spectrum of peptide **17**. Measured in positive ESI mode. The detected molecular weight was 1822.87. Chemical Formula: C81H122N20O28, Exact Mass: 1822.8737, Molecular Weight: 1823.9790.

Figure **S38**. Analytical chromatogram of peptide **17**.

**Peptide 18 –** Mouse preptin fragment.

FPRYPVGKFFQYNTW-amide

**Figure S39.** Deconvoluted mass spectrum of peptide **18**. Measured in positive ESI mode. The detected molecular weight was 1948.9638. Chemical Formula: C98H128N22O21, Exact Mass: 1948.9624, Molecular Weight: 1950.2350.

Figure **S40**. Analytical chromatogram of peptide **18**.

**Peptide 19 –** Rat preptin

DVSTSQAVLPDDFPRYPVGKFFKFDTWRQSAGRL


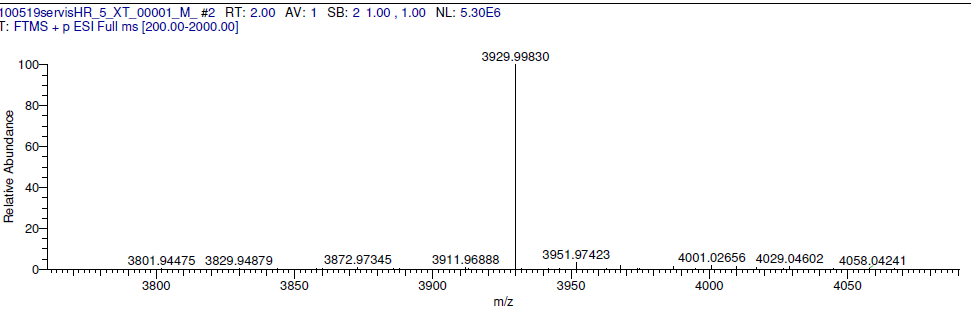


**Figure S41.** Deconvoluted mass spectrum of peptide **19**. Measured in positive ESI mode. The detected molecular weight was 3929.9983. Chemical Formula: C181H268N48O51, Exact Mass: 3929.9853, Molecular Weight: 3932.4200.

Figure **S42**. Analytical chromatogram of peptide **19**.

# NMR configuration of stereochemistry

**Table S1**. Proton and carbon-13 chemical shifts of peptide **10**.

(^1^H at 600 MHz; ^13^C at 150.9 MHz and ^15^N at 60.8 MHz; in H_2_O + D_2_O 95:5 + CD_3_COOD; pH=3.0; T = 25 deg;

chemical shifts are referenced to ^1^H and ^13^C signal of methyl group in AcOD using δH = 2.05 and δC = 22.75;

^15^N chemical shift are referenced to external CH_3_NO_2_ using δN = 381.7).

| **Residue** | **NH** | **Hα** | **Hβ** | **Hγ** | **Other protons** |
| --- | --- | --- | --- | --- | --- |
| **Thr-1** | *^a^* | 3.90 | 4.13 | 1.34 | -- |
| **Trp-2** | 8.93 | 4.74 | 3.34; 3.20 | -- | 10.26 (N1H), 7.25 (H2), 7.73 (H4), 7.19 (H5), 7.27 (H6), 7.52 (H7) |
| **Xxx-3 [R]** | 7.28 | -- | 1.37 | 1.00; 0.62 | 1.81 (Hδ); 5.21 (Hε) |
| **Gln-4** | 7.80 | 4.26 | 2.11; 1.90 | 2.30 | 7.47 and 6.87 (NεH_2_) |
| **Ser-5** | 8.13 | 4.39 | 3.92; 3.89 | -- | -- |
| **Thr-6** | 7.75 | 4.22 | 4.25 | 1.18 | -- |
| **Xxx-7 [R]** | 8.16 | 4.44 | 2.88; 2.74 | -- | 3.02 and 2.98 (Hδ); 5.34 (Hε) |
| **Arg-8** | 8.48 | 4.40 | 1.86; 1.75 | 1.63; 1.57 | 3.18 (Hδ), …. (NεH) |
| **Leu-9** | 8.24 | 4.30 | 1.65; 1.58 | 1.60 | 0.92 (Hδ1), 0.86 (Hδ2), 7.58 and 7.06 (CONH_2_) |

| **Residue** | **NH** | **CO** | **Cα** | **Cβ** | **Cγ** | **Other carbons** |
| --- | --- | --- | --- | --- | --- | --- |
| **Thr-1** | *^a^* | 170.86 | 61.57 | 69.22 | 21.81 | -- |
| **Trp-2** | 126.20; 129.42 *^b^* | 175.32 | 58.15 | 30.09 | -- | 127.72 (C2), 111.11 (C3), 121.34 (C4), 122.36 (C5), |
|  |  |  |  |  |  | 125.06 (C6), 115.00 (C7), 139.41 (C8), 129.38 (C9) |
| **Xxx-3 [R]** | 133.86 | 179.33 | 62.73 | 40.48 | 24. 29 | 33.53 (Cδ), 137.01 (Cε), 22.26 (CH_3_) |
| **Gln-4** | 115.62; 112.52 *^c^* | 177.14 | 56.55 | 29.34 | 34.25 | 180.68 (Cδ) |
| **Ser-5** | 115.30 | 174.58 | 59.29 | 63.89 | -- | -- |
| **Thr-6** | 111.76 | 174.56 | 62.30 | 69.51 | 21.92 | -- |
| **Xxx-7 [R]** | 123.49 | 174.83 | 57.07 | 32.85 | -- | 35.95 (Cδ), 128.67 (Cε) |
| **Arg-8** | 124.85 | 175.98 | 56.07 | 30.90 | 27.17 | 43.39 (Cδ), 159.73 (=C<) |
| **Leu-9** | 124.34; 107.71 *^c^* | 180.13 | 55.09 | 42.72 | 26.12 | 24.29 (Cδ1), 23.34 (Cδ2) |

*^a^* not detected; *^b^* Aryl-NH; *^c^* CONH_2_.

**Table S2**. Proton and carbon-13 chemical shifts of peptide **11**.

(^1^H at 600 MHz; ^13^C at 150.9 MHz and ^15^N at 60.8 MHz; in H_2_O + D_2_O 95:5 + CD_3_COOD; pH=3.0; T = 25 deg;

^1^H and ^13^C chemical shifts are referenced to signal of methyl group in AcOD using δH = 2.05 and δC = 22.75;

^15^N chemical shift are referenced to external CH_3_NO_2_ using δN = 381.7).

| **Residue** | **NH** | **Hα** | **Hβ** | **Hγ** | **Other protons** |
| --- | --- | --- | --- | --- | --- |
| **Thr-1** | *^a^* | 3.91 | 4.16 | 1.34 | -- |
| **Trp-2** | 8.93 | 4.80 | 3.34; 3.22 | -- | 10.25 (N1H), 7.25 (H2), 7.71 (H4), 7.19 (H5), 7.27 (H6), 7.53 (H7) |
| **Xxx-3 [R]** | 7.29 | -- | 1.36 | 1.00 | 1.86 and 1.78 (Hδ); 5.34 (Hε) |
| **Gln-4** | 7.73 | 4.32 | 2.10; 1.92 | 2.29; 2.10 | 7.45 and 6.87 (NεH_2_) |
| **Ser-5** | 8.30 | 4.35 | 3.98; 3.86 | -- | -- |
| **Thr-6** | 7.76 | 4.22 | 4.34 | 1.19 | -- |
| **Xxx-7 [S]** | 7.84 | 4.38 | 2.80; 2.78 | -- | 3.09 and 3.02 (Hδ); 5.30 (Hε) |
| **Arg-8** | 8.71 | 4.32 | 1.92; 1.76 | 1.69 | 3.21 (Hδ), …. (NεH) |
| **Leu-9** | 8.13 | 4.30 | 1.71; 1.65 | 1.62 | 0.92 (Hδ1), 0.87 (Hδ2), 7.44 and 7.06 (CONH_2_) |

| **Residue** | **NH** | **CO** | **Cα** | **Cβ** | **Cγ** | **Other carbons** |
| --- | --- | --- | --- | --- | --- | --- |
| **Thr-1** | *^a^* | 170.94 | 61.55 | 69.22 | 21.82 | -- |
| **Trp-2** | 125.98; 129.44 *^b^* | 175.37 | 58.15 | 30.06 | -- | 127.73 (C2), 111.12 (C3), 121.33 (C4), 122.34 (C5), |
|  |  |  |  |  |  | 125.03 (C6), 116.02 (C7), 139.39 (C8), 129.38 (C9) |
| **Xxx-3 [R]** | 133.69 | 179.14 | 62.75 | 40.59 | 24.32 | 33.60 (Cδ), 136.68 (Cε), 22.54 (CH_3_) |
| **Gln-4** | 115.74; 112.60 *^c^* | 177.24 | 56.09 | 29.18 | 34.22 | 180.72(Cδ) |
| **Ser-5** | 116.50 | 174.90 | 59.48 | 63.75 | -- | -- |
| **Thr-6** | 110.84 | 175.03 | 62.33 | 69.26 | 22.09 | -- |
| **Xxx-7 [S]** | 121.02 | 175.66 | 56.97 | 33.17 | -- | 35.68 (Cδ), 128.88 (Cε) |
| **Arg-8** | 125.15 | 176.37 | 56.56 | 30.58 | 27.42 | 43.40 (Cδ), 159.76 (=C<) |
| **Leu-9** | 122.66; 107.50 *^c^* | 180.27 | 55.18 | 42.50 | 27.24 | 25.08 (Cδ1), 23.34 (Cδ2) |

*^a^* not detected; *^b^* Aryl-NH; *^c^* CONH_2_.

# Table S3. Details of the CD experiments.

| **Peptide** | **N^a^** | **c^b^ (M.L^-1^)** | **M^c^ (g.mol^-1^)** |
| --- | --- | --- | --- |
| *human preptins and analogues* | | | |
| **1** | 34 | 2.5×10^-5^ | 4029.5 |
| **2** | 16 | 5.5×10^-5^ | 1818.0 |
| **3** | 18 | 4.5×10^-5^ | 2229.5 |
| **4** | 16 | 5.5×10^-5^ | 1817.0 |
| **5** | 34 | 2.5×10^-5^ | 4029.5 |
| **6** | 34 | 2.5×10^-5^ | 3986.5 |
| **7** | 34 | 2.5×10^-5^ | 3973.5 |
| **8** | 34 | 2.5×10^-5^ | 3974.4 |
| **9** | 9 | 8.5×10^-5^ | 1187.4 |
| **10** | 9 | 8.6×10^-5^ | 1159.4 |
| **11** | 9 | 8.8×10^-5^ | 1141.3 |
| **12** | 8 | 8.8×10^-5^ | 1045.2 |
| **13** | 9 | 8.7×10^-5^ | 1146.3 |
| ***mouse preptins and analogues*** | | | |
| **14** | 34 | 2.5×10^-5^ | 3948.4 |
| **15** | 34 | 3.0×10^-5^ | 3293.3 |
| **16** | 16 | 5.5×10^-5^ | 1809.9 |
| **17** | 16 | 5.5×10^-5^ | 1823.9 |
| **18** | 15 | 5.1×10^-5^ | 1949.2 |
| ***rat preptin*** | | | |
| **19** | 34 | 2.5×10^-5^ | 3932.4 |

^a^ Number of amino acid residues in a peptide; ^b^ molar concentration; ^c^ molar mass.

**Table S4.** Experimental CD band wavelengths l (nm) and intensities De (L.mol^-1^.cm^-1^)

| **Peptide** | **l** | **De** | **l** | **De** | **l** | **De** |
| --- | --- | --- | --- | --- | --- | --- |
| **1** |  |  | 200 | -3.8 |  |  |
| **2** |  |  | 200 | -3.4 |  |  |
| **3** |  |  | 198 | -2.0 |  |  |
| **4** |  |  | 200 | -4.2 |  |  |
| **5** |  |  | 199 | -2.8 |  |  |
| **6** |  |  | 203 | -2.9 |  |  |
| **7** | 192 | 0.9 | 206 | -2.4 | 222 | -1.2 |
| **8** |  |  | 203 | -2.9 |  |  |
| **9** |  |  | 198 | -2.1 | 222 | 0.6 |
| **10** |  |  | 191 | -1.8 | 221 | 1.4 |
| **11** |  |  | 199 | -2.5 | 224 | 0.8 |
| **12** |  |  | 198 | -2.6 | 224 | 0.1 |
| **13** |  |  | 198 | -4.1 | 227 | 0.1 |
| **14** |  |  | 201 | -3.5 |  |  |
| **15** |  |  | 199 | -2.5 |  |  |
| **16** |  |  | 197 | -3.8 |  |  |
| **17** |  |  | 196 | -3.5 |  |  |
| **18** |  |  | 191 | -2.5 | 228 | 0.3 |
| **19** |  |  | 200 | -4.3 |  |  |

**Table S5.** Secondary structure content (in %) estimated^a^ for peptides **1**-**19** according to the experimental CD.

| **Secondary**  **Structure** | **Peptides** | | | | | | | | | | | | | | | | | | |
| --- | --- | --- | --- | --- | --- | --- | --- | --- | --- | --- | --- | --- | --- | --- | --- | --- | --- | --- | --- |
|  | **1** | **2** | **3** | **4** | **5** | **6** | **7** | **8** | **9** | **10** | **11** | **12** | **13** | **14** | **15** | **16** | **17** | **18** | **19** |
| Helix (regular) | 1 | 2 | 0 | 1 | 1 | 4 | 6 | 4 | 0 | 0 | 0 | 0 | 0 | 1 | 1 | 0 | 0 | 0 | 0 |
| Helix (distorted) | 6 | 7 | 0 | 7 | 0 | 6 | 8 | 6 | 0 | 0 | 0 | 0 | 0 | 1 | 1 | 0 | 0 | 0 | 2 |
| Anti (left-twisted) | 0 | 0 | 0 | 0 | 0 | 0 | 0 | 0 | 0 | 0 | 0 | 0 | 0 | 0 | 0 | 0 | 0 | 0 | 0 |
| Anti (relaxed) | 9 | 8 | 17 | 9 | 14 | 10 | 10 | 11 | 16 | 20 | 14 | 17 | 14 | 13 | 14 | 13 | 14 | 18 | 9 |
| Anti (right-twisted) | 17 | 18 | 18 | 18 | 20 | 15 | 13 | 17 | 20 | 20 | 25 | 19 | 23 | 19 | 17 | 21 | 21 | 19 | 21 |
| Parallel | 0 | 4 | 0 | 5 | 1 | 1 | 2 | 0 | 0 | 0 | 0 | 0 | 0 | 0 | 0 | 0 | 0 | 2 | 0 |
| Turn | 15 | 14 | 16 | 14 | 16 | 15 | 15 | 15 | 17 | 19 | 15 | 16 | 17 | 17 | 17 | 17 | 16 | 16 | 17 |
| Others | 52 | 49 | 49 | 47 | 47 | 49 | 46 | 47 | 47 | 41 | 46 | 48 | 46 | 49 | 51 | 49 | 49 | 46 | 51 |
| **Grouped** |  |  |  |  |  |  |  |  |  |  |  |  |  |  |  |  |  |  |  |
| Helix | 7 | 8 | 0 | 8 | 2 | 10 | 14 | 10 | 0 | 0 | 0 | 0 | 0 | 2 | 2 | 0 | 0 | 0 | 2 |
| Antiparallel | 27 | 26 | 35 | 26 | 35 | 25 | 23 | 28 | 36 | 39 | 39 | 36 | 37 | 32 | 31 | 34 | 35 | 37 | 30 |
| Parallel | 0 | 4 | 0 | 5 | 1 | 1 | 2 | 0 | 0 | 0 | 0 | 0 | 0 | 0 | 0 | 0 | 0 | 2 | 0 |
| Turn | 15 | 14 | 16 | 14 | 16 | 15 | 15 | 15 | 17 | 19 | 15 | 16 | 17 | 17 | 17 | 17 | 16 | 16 | 17 |
| Others | 52 | 49 | 49 | 47 | 47 | 49 | 46 | 47 | 47 | 41 | 46 | 48 | 46 | 49 | 51 | 49 | 49 | 46 | 51 |

^a^ The secondary structure content of all proteins was estimated using the BeStSel program (4, 5).

# Preparation of mouse [^125^I]-Preptin ( derivative of peptide 14)

Radiolabeled [^125^I]-monoiodotyrosyl-preptin-1 was prepared via radioiodination of tyrosine moiety of mouse preptin **14** with ^125^I (Na[^125^I], product code: I-RB-41, IZOTOP, Hungary), using the Iodo-gen^TM^ system (Pierce oxidizing reagent).

An Eppendorf PP tube (1.5 mL), pre-coated in house with 10 nmoles of Iodo-gen^TM^ (Pierce), was rinsed with 200 μL of PBS (0.2 M phosphate buffer pH 8.0). Fresh PBS (200 μL), sodium iodide (5 μL Na[^125^I], 0.5 mCi, 0.25 nmol) and aqueous solution of the preptin **14** (100 μL, 1 μg/μL, 25 nmol) were mixed in the reaction tube. The tube was vigorously shaken for 15 min at room temperature and then 100 μL of 20 mM HEPES buffer (pH 7.3) containing BSA (1 mg/mL) was added. BSA (void of interfering IGF-like binding proteins, product code: A6003, Sigma) was used to suppress the stickiness of the peptide to the reaction tube walls. The mixture was directly injected into the semipreparative radio-HPLC system (Waters Alliance e2695, 2995 PDA detector and Empower 3 software for processing of data) combined with radioactivity-HPLC flow detector Ramona Star (Elysia-Raytest, Germany). The desired fraction containing [^125^I]-monoiodotyrosyl-preptin **14** was separated from the unmodified and over-iodinated analog on Nucleosil 120 C18 column (5μ, 250 × 4.0 mm, Watrex) at 25 °C. The mobile phases were: A, 0.1 % (v/v) TFA in 100 % water and B, 0.1 % (v/v) TFA in 100 % acetonitrile. The flow rate was 1 mL/min. The gradient started at 20 % mobile phase B, increased linearly to 30 % B over 30 min. Fractions were collected to tubes with 200 μL of BSA cocktail (12.5 mM sodium phosphate pH 7.4, 50 mM NaCl, 0.25 M glycine and 0.06 % BSA). The isolated fractions containing [^125^I]-preptin **14** were transferred to LoBind® Eppendorf tubes and evaporated to dryness on CentriVap at room temperature for 3 hours. The non-iodinated analog and three fractions of iodinated product were separated. Radioactivity was measured using the Gamma Counter Wizard 2470 (Perkin Elmer). The retention time of the unmodified analog was 26 min. The desired fraction of mono-iodinated product had a retention time of 34 min. We finally isolated 72 μCi (2.7 MBq) of mono-iodinated preptin (SA = 2100 Ci/mmol, 15.0 % RCY, >99 % RCP).

**Equipment:** Analytical-semipreparative radio-HPLC (Waters Alliance e2695, 2995 PDA detector and Empower 3 software for processing of data) combined with radioactivity-HPLC flow detector Ramona Star (Elysia-Raytest). The Gamma Counter Wizard^2^ 2470 (Perkin Elmer) for quantification of γ-ionization.

**
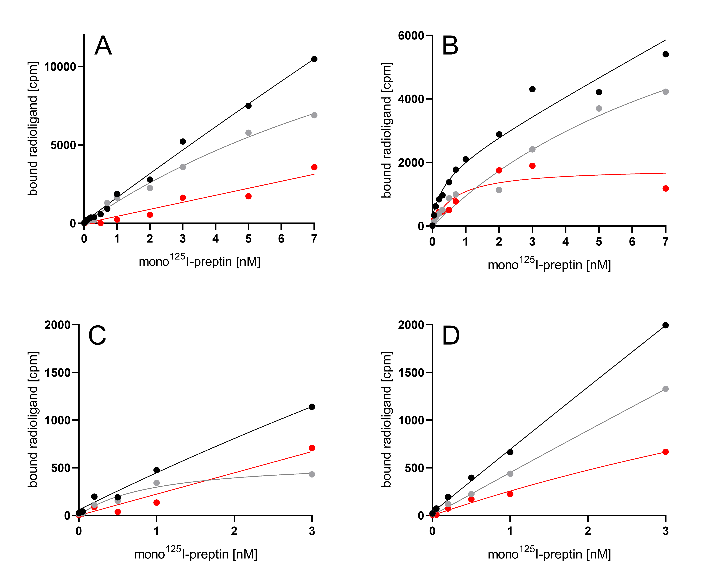
**

**Figure S43.** Typical saturation binding curves of [^125^I]-preptin **14** to R- cells (**A**), MC3T3-E1 cells (**B**), U-2 OS cells (**C**), MIN6 cells (**D**). The curves representing specific binding are colored in red, non-specific binding is shown in grey and total binding in black.


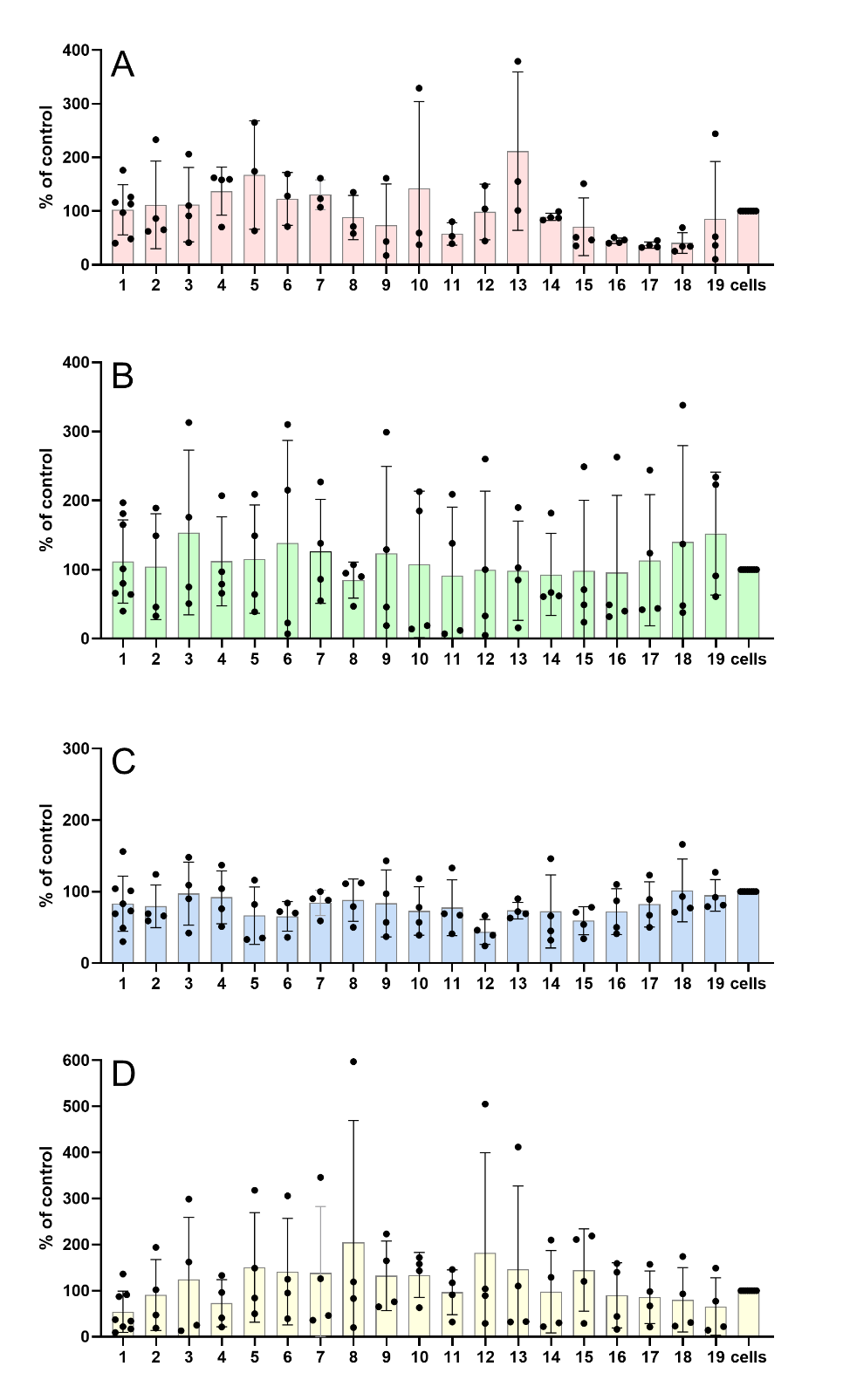


**Figure S44.** Stimulation of phosphorylation of Erk 1/2 by preptin derivatives **1**-**19**. Stimulation of phosphorylation of intracellular Erk 1/2 proteins in murine fibroblasts with deleted *Igf1r* gene (R- cells) (**A**), in MC3T3-E1 preosteoblasts (**B**), in U-2 OS osteoblast (**C**) and MIN6 pancreatic beta cells (**D**) by respective preptins or preptin analogues. Data are presented as means ± S.D., relative to the signal in non-stimulated cells. No significant differences were found between controls (cells) and peptides (determined using Ordinary one-way ANOVA).


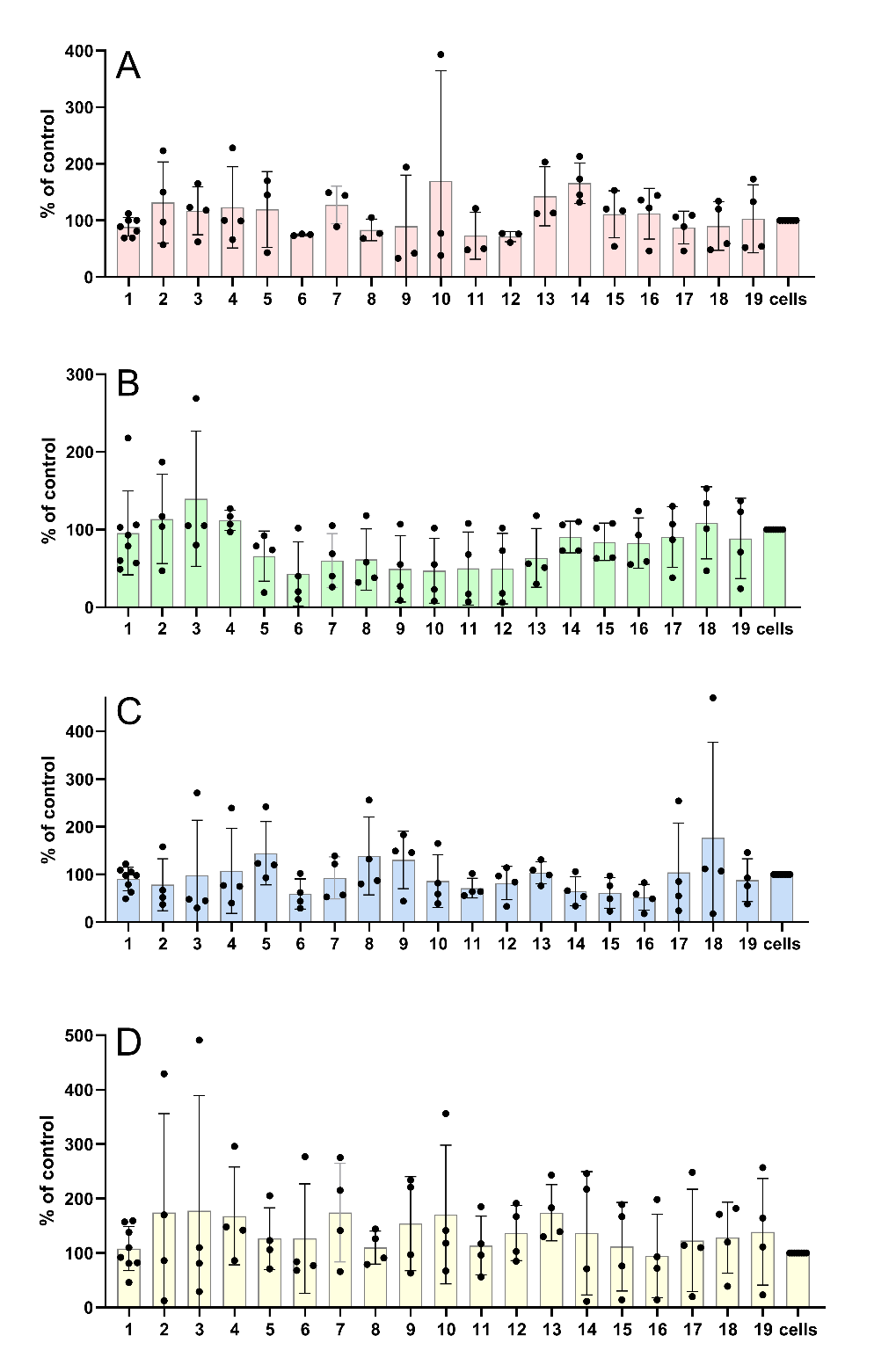


**Figure S45.** Stimulation of phosphorylation of PI3K p110α by preptin derivatives **1**-**19**. Stimulation of phosphorylation of intracellular PI3K p110α proteins in murine fibroblasts with deleted *Igf1r* gene (R- cells) (**A**), in MC3T3-E1 preosteoblasts (**B**), in U-2 OS osteoblast (**C**) and MIN6 pancreatic beta cells (**D**) by respective preptins or preptin analogues. Data are presented as means ± S.D., relative to the signal in non-stimulated cells. No significant differences were found between controls (cells) and peptides (determined using Ordinary one-way ANOVA).


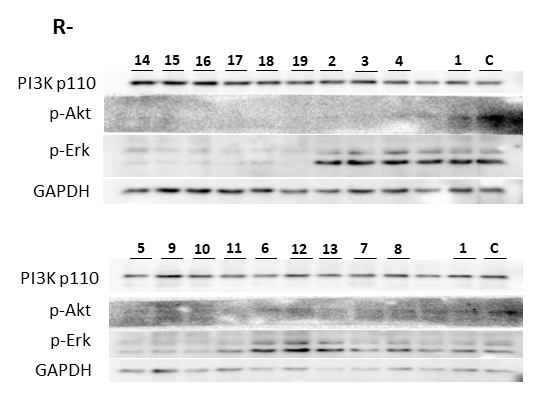


A


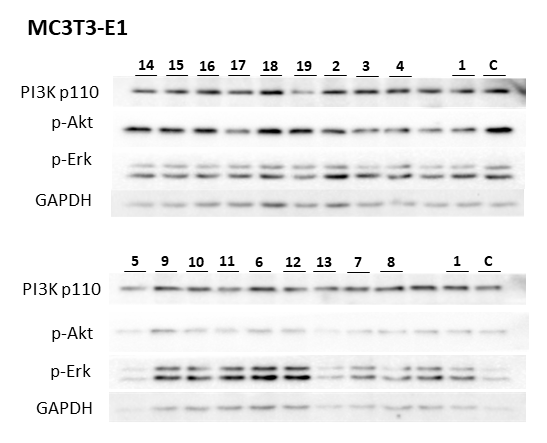


B


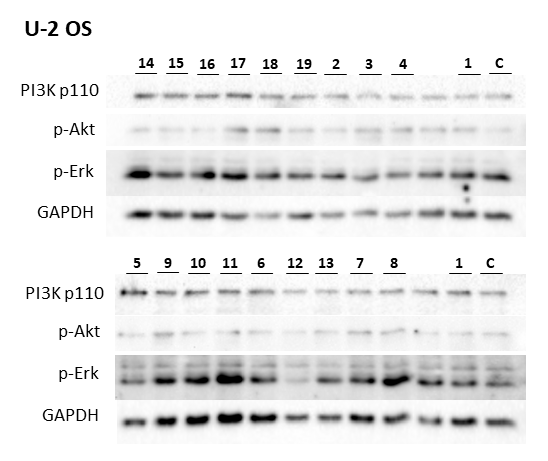


C


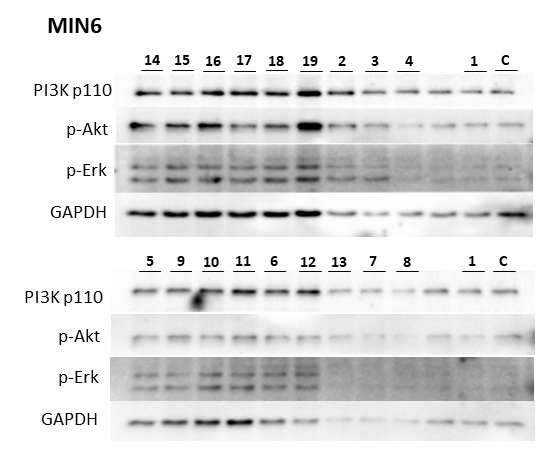


D

**Figure S46.** Representative western blot analysis of preptin derivatives **1-19**. Stimulation of phosphorylation of intracellular PI3K p110α, p-Akt and p-Erk proteins in murine fibroblasts with deleted *Igf1r* gene (R- cells) (**A**), in MC3T3-E1 preosteoblasts (**B**), in U-2 OS osteoblast (**C**) and MIN6 pancreatic beta cells (**D**). Stimulation was done by 10 nM IGF-1 for 10 min (see Methods). GAPDH were used as loading controls.

**References**

1. Góngora-Benítez M, Mendive-Tapia L, Ramos-Tomillero I, Breman AC, Tulla-Puche J, Albericio F. Acid-Labile Cys-Protecting Groups for the Fmoc/Bu Strategy: Filling the Gap. Org Lett. 2012;14(21):5472-5.

2. Machackova K, Collinsova M, Chrudinova M, Selicharova I, Picha J, Budesinsky M, et al. Insulin-like Growth Factor 1 Analogs Clicked in the C Domain: Chemical Synthesis and Biological Activities. J Med Chem. 2017;60(24):10105-17.

3. Behrendt R, Huber S, Marti R, White P. New-butyl based aspartate protecting groups preventing aspartimide formation in Fmoc SPPS. J Pept Sci. 2015;21(8):680-7.

4. Micsonai A, Moussong E, Wien F, Boros E, Vadaszi H, Murvai N, et al. BeStSele: webserver for secondary structure and fold prediction for protein CD spectroscopy. Nucleic Acids Res. 2022;50(W1):W90-W8.

5. Micsonai A, Wien F, Bulyaki E, Kun J, Moussong E, Lee YH, et al. BeStSel: a web server for accurate protein secondary structure prediction and fold recognition from the circular dichroism spectra. Nucleic Acids Res. 2018;46(W1):W315-W22.

1. [↑](#footnote-ref-1)
